# Glutamate Mediated Astrocytic Filtering of Neuronal Activity

**DOI:** 10.1371/journal.pcbi.1003964

**Published:** 2014-12-18

**Authors:** Gilad Wallach, Jules Lallouette, Nitzan Herzog, Maurizio De Pittà, Eshel Ben Jacob, Hugues Berry, Yael Hanein

**Affiliations:** 1 School of Electrical Engineering, Tel Aviv University, Ramat Aviv, Israel; 2 Team Beagle, INRIA Rhone-Alpes, Villeurbanne, France; 3 University of Lyon, LIRIS UMR5205, Villeurbanne, France; 4 Department of Electrical and Electronic Engineering, University of Nottingham, Nottingham, United Kingdom; 5 School of Physics and Astronomy, Tel Aviv University, Ramat Aviv, Israel; 6 Center for Theoretical Biological Physics, Rice University, Houston, Texas, United States of America; Université Paris Descartes, Centre National de la Recherche Scientifique, France

## Abstract

Neuron-astrocyte communication is an important regulatory mechanism in various brain functions but its complexity and role are yet to be fully understood. In particular, the temporal pattern of astrocyte response to neuronal firing has not been fully characterized. Here, we used neuron-astrocyte cultures on multi-electrode arrays coupled to Ca^2+^ imaging and explored the range of neuronal stimulation frequencies while keeping constant the amount of stimulation. Our results reveal that astrocytes specifically respond to the frequency of neuronal stimulation by intracellular Ca^2+^ transients, with a clear onset of astrocytic activation at neuron firing rates around 3-5 Hz. The cell-to-cell heterogeneity of the astrocyte Ca^2+^ response was however large and increasing with stimulation frequency. Astrocytic activation by neurons was abolished with antagonists of type I metabotropic glutamate receptor, validating the glutamate-dependence of this neuron-to-astrocyte pathway. Using a realistic biophysical model of glutamate-based intracellular calcium signaling in astrocytes, we suggest that the stepwise response is due to the supralinear dynamics of intracellular IP_3_ and that the heterogeneity of the responses may be due to the heterogeneity of the astrocyte-to-astrocyte couplings via gap junction channels. Therefore our results present astrocyte intracellular Ca^2+^ activity as a nonlinear integrator of glutamate-dependent neuronal activity.

## Author Summary

Over the past 20 years, astrocytes, a type of brain cells that were considerably disregarded, have gradually been found to actually display remarkable properties. In contrast with neurons, which communicate through changes in their membrane potential, astrocytes communicate as networks through propagated changes in their internal calcium concentration. While still hotly debated, recent findings even indicate that astrocytic activity could influence neuronal activity in several ways, including the modulation of synaptic plasticity between neurons. How exactly these astrocytes react to neuronal activity as individual cells and as a network is however still unclear. In this work, we address this question by using neuron-astrocyte cell cultures that we stimulate with multi-electrode arrays in conjunction with a computational model of neuron-astrocyte communication. Our results show that astrocytes respond to neuronal activity in a frequency-dependent manner through glutamate signaling. We also propose that the heterogeneity of astrocyte response time and oscillation frequencies that emerges from our experiments could be linked to their network organization. Since astrocytes are known to communicate with neurons by releasing signaling molecules upon activation, our findings, by shedding new light on astrocyte responses to neuronal activity, help uncover the complex mechanisms underlying neuron-astrocyte cross-talk.

## Introduction

Evidence obtained during the last few years inaugurated the notion that astrocytes may play a role in information processing in the brain. The "tripartite synapse" concept (presynaptic, postsynaptic, astrocyte) [1], [2] is gaining acceptance, and is progressively replacing the historical bipartite synaptic view (restricted to presynaptic and postsynaptic). This concept builds on expansive experimental evidence which shows that astrocytes display a form of excitability based on elevations of their intracellular Ca^2+^ concentrations ([Ca^2+^]_i_) in response to synaptically released neurotransmitters [3]–[8].

As astrocytes form non-overlapping domains, and can cover areas containing hundreds of dendrites (up to 140,000 synapses per a single astrocyte) [9], astrocytes are well positioned to propagate neuronal information to neighboring synapses, thus bypassing or enforcing the neuron-neuron pathway [1], [10]–[12].

The study of astrocyte-neuron communication is complicated by its bidirectional nature. Glutamate transmission is a typical example: just like neurons, astrocytes express efficient glutamate transporters that clear glutamate from the synaptic cleft and glutamate receptors (metabotropic); they also release glutamate in a process that may be similar to neurons [1], [13]. This suggests that neurons can transmit glutamate signals to astrocytes (feedforward communication) and vice-versa (feedback). Whether astrocytic glutamate release is regulated and able to transmit feedback signals from astrocytes to neurons in physiological conditions is still hotly debated [14]–[16]. On the contrary, the existence of the forward signaling, whereby neurons transmit glutamate-mediated signals to astrocytes, is well established [1], [17], [18]. Experimental evidence generally supports the idea that astrocytes are more than simple passive (linear) read-out of the synaptic activity but process it in an integrated and complex fashion, encoding the input neuron activity as a nonlinear response in their Ca^2+^ dynamics [13], [19]. However, how exactly astrocytes integrate and process synaptic information is still unclear. Thus, additional studies are needed to reveal the complex nature of neuron-to-astrocyte communication and the properties of astrocytic [Ca^2+^]_i_ signals evoked by synaptic activity.

In this study we focus on the feedforward glutamate signaling (from neurons to astrocytes) and show that neuron-to-astrocyte signaling includes a form of complex encoding and information processing, which was classically considered to be exclusively within the domain of neurons.

To explore neuronal modulation of astrocytic activity at a network level we used neuron-glia mixed cultures. We applied direct electrical stimulation with micro electrodes to selectively activate neurons while optically reading neuronal and astrocytic activation using a calcium imaging technique [20]. In particular, our setup features the possibility to independently set the frequency and the amount of neuronal stimulation. This property allows to unambiguously question which parameter of the neuronal stimulation (frequency or amount) is important for the astrocyte response. We show that astrocyte response is a nonlinear function of neuronal stimulation frequency, with an onset that varied from one astrocyte to the other between 1 and 10 Hz. This response was abolished by the application of metabotropic glutamate receptor antagonists, thus demonstrating that this signal is mediated by glutamate. Using a realistic biophysical model of glutamate-based intracellular calcium signaling in astrocytes, we suggest that the observed stepwise response is due to the supralinear dynamics of intracellular IP_3_ and that the heterogeneity of the responses may be due to the heterogeneity of the astrocyte-to-astrocyte couplings via gap junction channels. The results presented in this work thus indicate the existence of a rate dependent encoding process underlying neuro-glia pathway.

## Methods

### Preparation and growth of cultured networks

Dissociated cortical cultures were prepared from surgically removed cortices of E18 Sprague Dawley rat embryos. The cortical tissue was digested by 0.065% trypsin (Biological Industries, Beit Haemek, 03-046-1) in phosphate-buffered saline (Beit Haemek, 02-023-1) for 15 min, followed by mechanical dissociation by trituration. Cells were re-suspended in a modified essential medium (MEM) without phenol red nor glutamine (Gibco, 21200-046), complemented with 5% horse serum (Beith Haemek 04-004-1), 5 mg⋅ml^−1^ gentamycin (Beith Haemek 03-035-1), 50 µM glutamine (Beith Haemek 03-020-1) and 0.02 mM glucose (BDH101174Y). Cells were then plated on multielectrode arrays (MEAs) (500/30iR-Ti or HD 30/10iR-ITO, by Multi Channel Systems) coated by poly-D-lysine (PDL, Sigma, catalog no. p-7889), at density of 3500–4500 mm^−2^ (that is ∼2×10^6^ cells per culture). Cultures were maintained at 37°C with 5% CO_2_ at 95% of humidity. The growth medium was partially replaced every 3–4 days (approximately 30%).

### Pharmacology

Suppression of synapse efficacy was obtained by adding ∼1 µM b-cyano-7-nitroquinoxaline-2,3-dione (CNQX) which is a α-amino-3-hydroxy-5-methyl-4-isoxazelepropionic acid receptor (AMPAR) antagonist, and ∼3 µM (2R)-amino-5-phosphonovaleric acid (APV) which is a N-methyl-D-aspartate receptor (NMDAR) antagonist to the recording medium. This approach was shown to reduce functional connectivity in neuronal cultures [21]. Neuronal action potentials were blocked by 1.5 µM of the sodium channel blocker tetrodotoxin (TTX). Inhibition of astrocytic metabotropic glutamate receptors mGluR5 and mGluR1 was achieved by adding to the bath 25 µM 6-Methyl-2-(phenylethynyl)pyridine hydrochloride (MPEP) and 50 µM (S)-(+)-a-amino-4-carboxy-2-methylbenzeneacetic acid (LY367385) respectively [22]. All chemicals were purchased from Sigma-Aldrich.

### Immunocytochemistry

Cultures were washed twice in phosphate buffered solution (PBS), then fixed by 4% paraformaldehyde (Merck) solution for 10 min, and left in PBS before staining. To perform immunocytochemical staining, fixed cultures were washed three times with PBS (10 min/wash) and permeabilized by 0.5% triton X-100 (Sigma-Aldrich) in PBS for 10 min. Cultures were then blocked with 2% BSA, 10% normal donkey serum and 0.5% triton X-100 solution in PBS for 1 hr at room temperature and incubated overnight at 4°C with primary antibodies GFAP (1∶400 Sigma-Aldrich) and NeuN (1∶200, Millipore). Cultures were further washed by PBS (3 times, 10 min/wash) and incubated for 1 hr at room temperature with the appropriate secondary antibodies: Alexa fluor 488 goat anti rabbit IgG (1∶400, Jackson) for the detection of GFAP, and Cy-3 donkey anti-mouse IgG (1∶700, Jackson) for NeuN. Finally, after another wash by PBS (3 times, 10 min/wash), cultures were mounted with aqueous DAPI-containing medium (VECTASHIELD Mounting Medium with DAPI, Vector Laboratories, H-1200).

### Electrophysiology and Ca^2+^ imaging

Rectangular and biphasic 400 µs-long current pulses of 25–35 µA were applied to cell cultures by an extracellular multi-electrode array (MEA), using 30 µm diameter electrodes and a dedicated 4-channel stimulus generator (STG 2004, Multi Channel Systems). Note that for the study of cellular safety and efficacy of electrical stimulation, we express below the stimulus in units of charge density (mC/cm^2^), rather than current (µA), in order to normalize the electrode diameter and duration of stimulation pulses, thus enabling generic measures and facilitating comparisons.

Ca^2+^ imaging was performed in open air environment, and accordingly culture medium was replaced by buffered-ACSF medium (10 mM HEPES, 4 mM KCl, 2 mM CaCl^2^, 1 mM MgCl^2^, 139 mM NaCl, 10 mM D-glucose, adjusted with sucrose to an osmolarity of 325 mOsm, and with NaOH to a pH of 7.4). Cultures were washed three times to remove traces of incubation medium and incubated in ACSF with 3 µM Oregon-Green BAPTA-I (Invitrogen 06807, one vial with 6.7 µL Anhydrous-DMSO for stock of 6 mM) and same volume of Pluronic acid F-127 (Biotium 59000, stock 10% w/v after mixing 1 g vial in 10 ml distilled deionized water) for 30 min. Following incubation, cultures were washed again and kept in ACSF. During recordings, cultures were kept at 37°C. Time lapse data were taken with an Olympus upright microscope (BX51WI) fitted with an EMCCD camera (Andor Ixon-885) and a ×20 water immersion objective (Olympus, UMPLFLN 20XW NA 0.5). This setup allowed the visualization of cells residing on top of non-transparent electrodes. Fluorescent excitation was delivered by a 120 W mercury lamp (EXFO x-cite 120PC) coupled with a dichroic mirror with a filter to match the dye spectrum (Chroma T495LP). Camera control utilized Andor propriety SOLIS software. Time-lapse recordings were performed at 2×2 binning mode for resolution of 500×502 and 51.948 frames per second. Time lapse sequences were collected via a dedicated 12 bit Andor data acquisition card installed on a personal computer, spooled to a high capacity hard drive and stored as uncompressed multi-page TIFF file libraries. The effect of bleaching was very moderate and addressed by using normalization of fluorescence values (ΔF/F_0_). A scheme illustrating our experimental setup is given as S1 Fig.

In cell cultures at the culture stage observed in this paper (DIV 14–27), neural activity typically organizes as periodic synchronized bursting events in which most neurons fire once or several times within a short time interval (population bursts) [23]–[25]. The amplitude and extension of these bursts of neural activity are so large that they eclipse the subtler crosstalk between neurons and glial cells. To overcome this issue and focus on the neuron-glia interactions, we used synaptic blockers APV and CNQX (see “Pharmacology”) which suppress the spontaneous bursting events. Each Ca^2+^ imaging session typically consisted in the observation of 3–4 different fields of view per each neuron-astrocyte culture dish.

### Analysis of Ca^2+^ data

Ca^2+^ imaging data was stored as uncompressed TIFF library, where pixel values represented fluorescence intensity. Boundaries of astrocyte somata were semi-automatically segmented from the time-averaged Ca^2+^ image using a custom code implemented in MATLAB (The MathWorks Inc., Natick, Massachussetts, USA), followed by manual adjustments. Ca^2+^ variations in the astrocyte cell bodies were estimated as normalized changes of fluorescence signal from baseline(ΔF/F_0_). Local baseline fluorescence (F_0_) was evaluated from the histograms of the signal within a running time window. Time windows without cellular activity were best fit by a single Gaussian (due to white noise), whereas those with cellular activity were best fit by two Gaussians (due to white noise and activity). For display purposes, the signal was smoothed by convolution with a 50-data point-large Savitsky-Golay filter of polynomial degree 7 [26], [27]. Neuronal signals were distinguished from astrocytic signals based on typical dynamic time scales, and physiological properties of their calcium signals [28]. Neurons were characterized by fast variations in Ca^2+^ activity during spike onset, whereas astrocytes exhibited slowly varying signals (S2 Fig.).

Neuronal activation probability was calculated over several stimulations at different amplitudes. The probability is defined for each neuron as the portion of times it responded by an action potential to an electrical stimulation at a specific amplitude. We neglected neurons with no response throughout the whole stimulation range. The stimulation threshold was defined for each neuron as the amplitude which activated it with 0.35 probability. For astrocytes, *single-cell* responsiveness to electrical stimulation at a given frequency was estimated by the sum of fluorescence values Δ*F*/*F*_0_ for all time windows at which electrical stimulation was applied to a given astrocyte, and normalized by the length of the time window. The spontaneous responsiveness of the astrocyte, calculated as the fluorescence values Δ*F*/*F*_0_ recorded in absence of stimulation, was then subtracted, to eliminate the contribution of spontaneous activity to the measured responsiveness. In other words, the responsiveness *r* was computed as
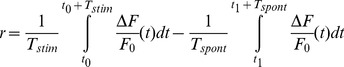
(1)where *t*_0_ is the start of the stimulation, *T*_stim_ its duration, and *t*_1_ and *T*_spont_ are the same parameters but in the absence of stimulation. Note that *r* has no dimension and will be given below in arbitrary units (AU). An astrocyte was considered to be *stimulated* by neuronal activity when its recorded Ca^2+^ response was highly correlated in time with the applied electrical stimulation. *Population* responsiveness to electrical stimulation at a given frequency was defined as the average of the single-cell responsiveness of all the stimulated astrocytes in a given experiment.

Single-cell and population responsiveness as a function of the frequency of electrical stimulation were generally sigmoid in shape, with an exponential rising phase. To compute the onset frequency, that is the smallest frequency of the electrical stimulation that triggered a detectable astrocyte response, the responsiveness (at the single-cell or population level, as indicated in the text or captions) was fitted by a four-parameter logistic function:
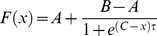
(2)where *A* is the minimum plateau, *B* is the maximum plateau, *C* is the 50% point, and τ controls the maximum slope. In cases where the average responsiveness did not reach a plateau for the largest applied frequency, the sigmoid fit was poor, and therefore we interpolated the results to the exponential growth part of the function. In all cases, the onset frequency was defined using the fitted function as the frequency for which the astrocyte responsiveness was 10% of its maximum value. Error was quantified according to the fits 95% confidence level. We used this definition of onset frequency for both experimental data and their simulated counterparts since they were associated to a small number of different stimulation frequencies. Fitting a sigmoid function thus allowed us to interpolate between these few points and estimate the onset frequency. For specific simulations that were independent from experimental data, we were no longer limited by the restricted number of data points and we thus computed the *exact onset frequency* as the stimulation frequency corresponding to the inflection point of the responsiveness curve.

Astrocytic intracellular Ca^2+^ traces form a very non-stationary signal. Therefore, to quantify astrocyte-oscillating frequencies during electrical stimulation, we applied time-frequency analysis. Wavelet decomposition [29]–[32] was applied on each Ca^2+^ trace. Compared to the more classical methods based on short-term Fourier transforms [33], wavelet decomposition provides a better tradeoff between time and frequency resolution and does not require setting a specific sliding window length. We used the Morlet wavelet basis, which is composed of a complex exponential (carrier) multiplied by a Gaussian window (envelope) as its shape resembled that of astrocytic Ca^2+^ signals. Signal pseudo-frequencies in Hz were calculated according to the scale parameter using the wavelet center frequency, defined as the highest amplitude in the Fourier transform of the Morlet function. In order to extract only the significant astrocyte oscillations, we restricted the time-frequency plane to those time points that were within the electrical stimulation windows and which had at least one transform coefficient greater than 70% of the largest transform coefficient. For each astrocyte and each time point in this restricted time-frequency plane, we extracted the representative frequency of this astrocyte at this time point as the frequency that showed the largest transform coefficient at that time point. We then built for each astrocyte the distribution of its representative frequencies. Finally, we defined the *maximal oscillating frequency* of an astrocyte as the 95 percentile of the distribution of its representative frequencies (we could not use the maximum of the distribution because of potential artifacts from the wavelet transform).

### Model astrocytic Ca^2+^ dynamics

**Figure 1:**
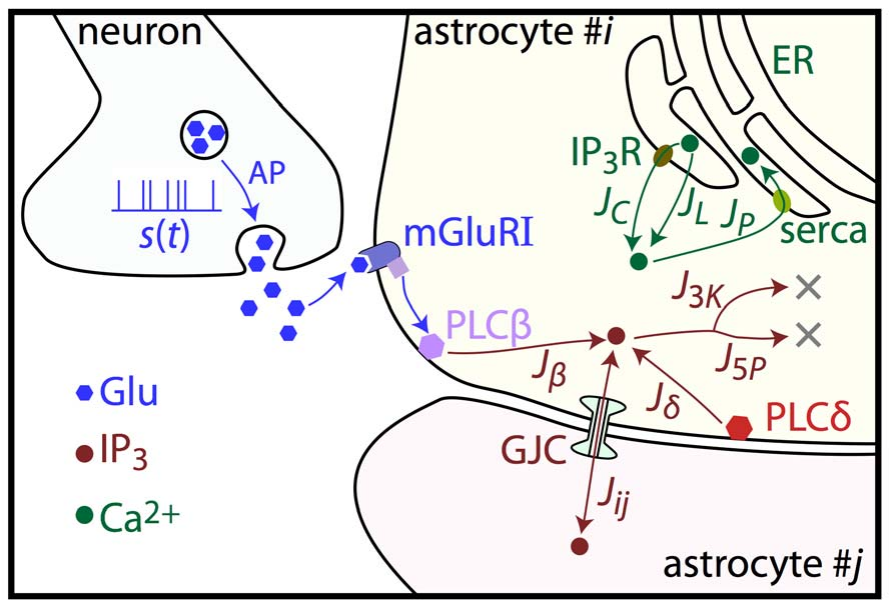
The G-ChI model for glutamate-stimulation of intracellular calcium dynamics in astrocytes. This graphical model introduces and summarizes the main variables and rate processes accounted for by the model. The stimulating neuron emits action potentials at times points given by the sequence *s*(*t*). The corresponding amount of released glutamate (Glu) is computed under the hypothesis of a dynamic synapse (Tsodyks-Markram model). Glutamate then activates group I metabotropic receptors (mGluRI) on the astrocyte membrane, thus activating Phospholipase Cβ (PLCβ), which results in the formation of IP_3_ with rate *J_β_*. IP_3_ is also synthesized with rate *J_δ_* by calcium-activated Phospholipase Cδ (PLCδ) and transformed to other metabolites by two enzymes: IP_3_ 3-kinase (rate *J_3K_*) and inositol polyphosphate 5-phosphatase (rate *J_5P_*). IP_3_ is also transported to other astrocytes to which the astrocyte is coupled via Gap Junction Channels (GJC) (flux *J_ij_*). The model moreover accounts for Calcium-Induced-Calcium-Release (CICR): Ca from intracellular stores (mostly the Endoplasmic Reticulum, ER) is pumped into the cytoplasm by Ca- and IP_3_-regulated IP_3_-Receptor Channels (IP_3_R, rate *J_C_*) of the ER membrane and reintegrated by sarco/endoplasmic reticulum Ca2+-ATPase (SERCA) pumps (rate *J_P_*). The model also accounts for passive IP_3_ leak through the ER membrane (rate *J_L_*). The equations describing this graphical model are outlined in the main text (Methods section). More information can be found in Ref [34] and [35].

Intracellular Ca^2+^ dynamics in the cytoplasm of astrocytes in response to glutamatergic neuronal stimulation can be described by the *G-ChI* model that we previously developed and studied, and provides a realistic description of Ca^2+^ dynamics in an isolated astrocyte [34]. This model neglects possible spatial non-homogeneities of the intracellular distribution of chemical species, and the intricate and complex shape of astrocytes, thus simplifying model astrocytes as perfectly stirred cells with spherical shapes. The *G-ChI* model considers both Ca^2+^ regulation by IP_3_-dependent calcium-induced calcium released (CICR), as well as IP_3_ dynamics resulting from PLCδ and PLCβ-mediated production and degradation both by IP_3_ 3-kinase (3K) and inositol polyphosphate 5-phosphatase (5P). Fig. 1 illustrates the main processes involved in the dynamics, as well as the associated rates or fluxes. The [Ca^2+^]_i_ dynamics for each astrocyte 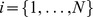
of the network are described by three coupled nonlinear ordinary differential equations:
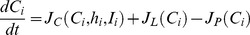
(3)

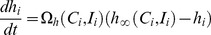
(4)

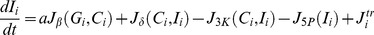
(5)


where the variables *C_i_*, *h_i_*, *I_i_*, *G_i_* respectively denote the cell-averaged cytosolic Ca^2+^ concentration, the fraction of activable IP_3_R channels on the endoplasmic reticulum (ER) membrane, the cell-averaged cytosolic IP_3_ concentration, and the glutamate concentration released by the presynaptic neuron in the extracellular space. In eq. (5), *a* indicates whether astrocyte *i* is directly activated by a synapse (in which case *a* = 1) or not (*a* = 0). The additional term 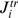
in equation (5) sums IP_3_ flows 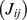
from/to any astrocyte *j* that is directly connected by gap junction channels (GJC-coupled) to astrocyte *i*, i.e. 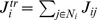 with 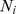 the set of astrocytes that are GJC-coupled to *i* [35]. See below for the modeling of astrocyte coupling via gap-junctions. Model parameters (S1 Table) were set as to display frequency modulation (FM) encoding ([35]) in order to match the shape of experimentally observed Ca^2+^ signals. Time-dependent parameters were then adjusted so that the maximum oscillation frequency of model astrocytes matched the maximum oscillation frequency (∼0.2 Hz) measured in our experiments. Additionally, since we did not witness intercellular Ca^2+^ waves in the experiments, we lowered the IP_3_ production rate of PLCδ so as to minimize intercellular waves in the model as well.

### Experimental-based astrocyte network modeling

**Figure 2:**
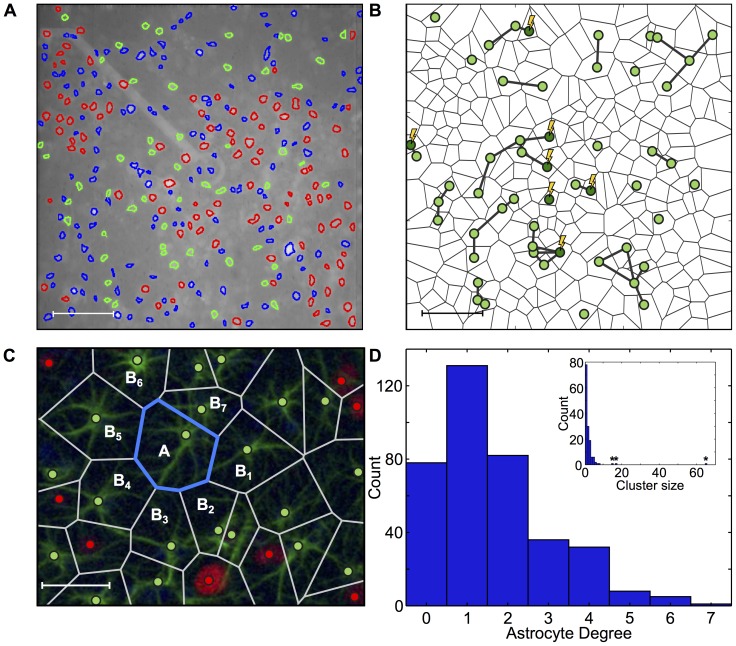
Inference of model networks from experimental data. **A**, The experimental culture of Fig. 2, with neurons segmented in *red*, astrocytes in *green* and unresponsive cells in *blue*. Scale bar is 75 µm. **B**, Model networks constructed using the experimental data of A. Fine *grey* lines delineate the Voronoi diagram computed from the experimental cell positions; *green* circles denote model astrocytes and *dark green* cells with a lightning symbol denote stimulated model astrocytes. Wide *dark grey* lines show the GJC connections between astrocytes. Scale bar is 75 µm. Model networks were inferred according to this process for each experiment. **C**, Close-up view of the Voronoi tesselation (*light gray* and *blue* lines) associated with an immunostaining image (in *red* the neuronal marker NeuN and in *green* the astrocytic marker GFAP). Astrocyte A will be GJC coupled to astrocytes B1 through B7 as they share boundaries of their anatomical domains (*thick blue* lines). **D**, Resulting distribution of astrocyte coupling degree. Most model astrocytes are connected to very few neighbors (*n* = 373). The inset shows the distribution of the size of connected astrocyte clusters (*n* = 146). It can be seen that most astrocytes are isolated but some experiments contain very large clusters of up to 60 astrocytes (indicated by stars).

As hinted by the presence of the 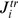 coupling term in equation (5), our model astrocytes were coupled to each other with gap-junction channels (GJC). For each computer simulation, we defined the coupling topology of the model astrocyte network on the basis of a corresponding cell culture, thus defining one-to-one correspondence between experiments and computer simulations. Cell cultures we composed of three types of cells: astrocytes, neurons, and unclassified cells (cells which did not show any Ca^2+^ activity). Since all these cells were constrained in a two-dimensional space and astrocytes are known to occupy separate anatomical domains [9], we assumed that GJC couplings were restricted to nearby astrocytes. For example, when two astrocytes were spatially separated by a neuron or an unclassified cell, we did not couple these two astrocytes in the model. To determine the precise coupling topology, cell positions were defined from the reference experiment as the center of mass of the cell bodies (retrieved as described above). The anatomical region of each cell was then established by computing the Voronoi diagram of the cells. For each cell, the Voronoi diagram actually associates the cell to a 2D region around it that is such that all points inside this region are closer to the center of its associated cell than to any other cell center. An illustration of a Voronoi diagram is given in grey (Fig. 2B) with its associated cell culture (Fig. 2A). All astrocyte pairs whose anatomical regions shared a border were GJC coupled in the model. As an illustration, Fig. 2C displays an immunostaining image together with its associated Voronoi tessellation (*light gray* lines); astrocyte A shares its anatomical region boundaries with 7 other astrocytes (B1 through B7). Assuming that all of its neighbors are correctly characterized as astrocytes (and not unclassified cells), A will be GJC linked to all its neighbors in the reconstructed network. Note that the non-astrocyte cells (and the astrocytes that were not classified as such because they did not display any Ca^2+^ activity) were not used subsequently in the model.

### GJC coupling

In terms of cell-averaged concentrations, the exchange of IP_3_ between two cells can be regarded as a multiscale phenomenon that depends on many factors, including astrocyte morphology and physiology, GJC location and permeability to IP_3_ [36]. To account for these factors, IP_3_ exchange between two GJC-coupled astrocytes *i* and *j* was assumed nonlinear. To model this nonlinearity, we chose to use a sigmoid function of the IP3 gradient between the two cells 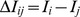, according to [35], [37]:
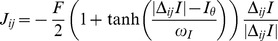
(6)


Where 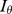 represents the threshold IP_3_ gradient for effective intercellular exchange, i.e. the minimal IP_3_ gradient for which *J_ij_*>0; *ω_I_* sets stiffness of this sigmoid function and *F* quantifies the strength of coupling between these two cells.

### Neuronal stimulation of the astrocyte network

Our computer model distinguishes two astrocyte populations: directly activated astrocytes, that receive direct glutamate stimulation from a neuronal process (for which *a* = 1 in eq. 5) and indirectly activated astrocytes (*a* = 0 in eq. 5) whose Ca^2+^ transients are triggered by a GJC-coupled neighbor astrocyte. For each astrocyte in the experiments and for each electrical stimulation frequency, we computed the time delay between the stimulation start and the initiation of the astrocyte Ca^2+^ response (defined as the smallest time after the stimulation start for which the normalized fluorescence goes over 60% of its maximum value). Every astrocyte showing in the associated experiment a time delay between stimulation start and initiation of the response of less than 1.5 s was defined as a *stimulated* astrocyte in the computer model. We chose this 1.5 s threshold in order to make sure that the Ca^2+^ responses of the stimulated astrocytes are due to neuronal stimulation and not to GJC coupled astrocytes. Indeed, even for the highest stimulation frequencies, astrocytes responded to the electrical stimulation in less than 1.5 s in the model whereas the typical delay needed to transmit a Ca^2+^ signal from one astrocyte to another via GJC coupling was 2s. Each stimulated astrocyte of the computer model was stimulated using the Tsodyks and Markram model [38], [39] for the average synaptic release in response to a sequence *s*(*t*) of action potentials (see S1 Text). The resulting model reads:
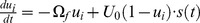
(7)

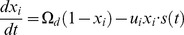
(8)

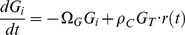
(9)where the product *r*(t) of the two synaptic variables *u* and *x*, that is 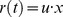
_,_ represents the fraction of synaptic glutamate released upon an action potential of the sequence *s* and *G_i_*(*t*) is the amount of glutamate in the synapse at time *t*. We assumed that each electrical pulse delivered by MEAs to the network triggered one action potential in each stimulating neuron, so that *s*(*t*) in the above equation actually represented the sequence of electrical stimulation of the MEA. Even in in vitro cultures, astrocytes extend processes that contact neurons [40]. However, as we cannot determine the number of synapses that contact a given stimulated astrocyte, we considered the above equations as a description of an equivalent synapse, accounting for all synapses enwrapped by one astrocyte.

Model parameters for all simulations are reported in S1 Table.

## Results

Mixed neuro-glia cultures from rat cortices on micro electrode array (MEA) substrates were used as a model experimental system to study neuro-glia communication. Optical recordings of intracellular Ca^2+^ levels were performed between 14 and 27 days in vitro (DIV) [20]. Neurons and astrocytes displayed spontaneous Ca^2+^ activity characterized by intracellular oscillations [41], [42] and short time windows of intense neuronal firing separated by longer intervals of sporadic firing. These neuronal network bursts (NBs) are typical of such cultures [23]–[25], and were even shown to intensify in cultures immersed in ACSF media such as the one used here for recording [43] (S3 Fig.). The amplitude and extension of these network bursts of neural activity are so large that they eclipse the subtler crosstalk between neurons and glial cells. To overcome this issue, our strategy was to silence the spontaneous network bursting activity by the application of NMDAR and AMPA/kainate receptor antagonists (AP-5 and CNQX respectively, see S4 Fig.) and apply electrical stimulation via the MEA electrodes to selectively elicit neuronal activity in a well-controlled manner. In those conditions, single cell analysis can be performed and shape analysis can easily be applied to distinguish between neuronal and astrocyte Ca^2+^ signals [20] (S2 Fig.).

### Mapping stimulation efficacy

**Figure 3:**
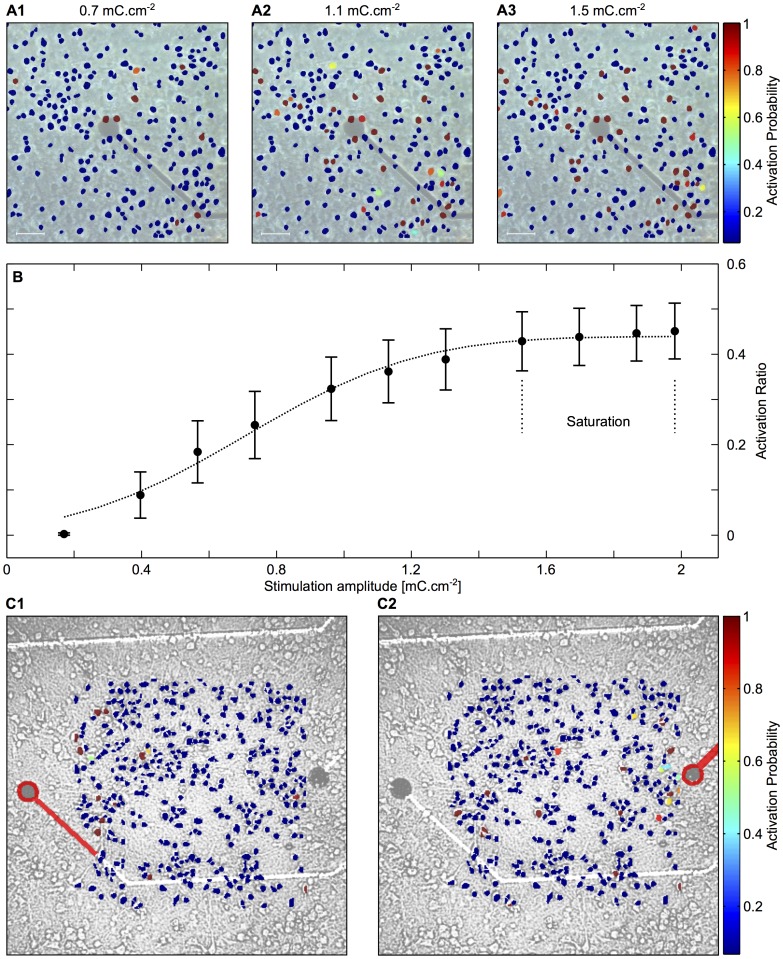
Geometric mapping of electrical activation. **A**, Response probability color coded activation maps for different stimulation amplitudes showing a clear correlation between stimulation amplitude and number of activated neurons (scale bar 50 µm). **B**, Proportion of activated neurons as a function of stimulation amplitude, indicating a saturation zone. **C**, Recordings incorporating stimulations from different electrodes provide insight regarding mechanism of electrical stimulation. Each electrode activates a unique set of neurons with a very small neuronal population that responds to both.

To validate our electrical stimulation conditions we first mapped the efficacy of MEA stimulations relative to the triggering of Ca^2+^ transients. To define a proper stimulation charge density range, we performed an activation safety mapping (S2C Fig.). Based on these experiments, we defined a maximal stimulation range of 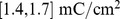. The stimulation efficacy was explored by mapping the location of activated neurons relative to the stimulating electrode. Fig. 3A shows neuronal activation maps at three different stimulation amplitudes (response probability is color coded). This figure shows that the distance between a neuron soma and the stimulation electrode is not highly correlated to the amplitude of the stimulation needed to activate it. Indeed, some of the neurons located far from the electrode are activated by lower stimulation amplitudes than neurons located close to the electrode. This suggests that activation is transmitted over long distances by the neuronal processes. This effect is further illustrated in Fig. 3B which shows the increase in the number of activated cells with increased charge density averaged over three cultures and eight electrodes. Activation ratio reaches a saturation level, suggesting that electrical stimulation activates processes at the electrode vicinity. Fig. 3C shows activation maps depicting the response to stimulations applied at two different electrodes (highlighted). Each electrode activated a unique set of neurons and the neuron population that responded to both electrodes was very small. This confirms that the distance between neuron and electrode is not a dominant factor determining response probability. Altogether these results indicate that in our MEA setup, electrical neuronal activation is dominated by the activation of neuronal processes rather than by that of the soma and is highly non-localized [44], [45].

### Astrocyte activation by neuronal activity is mediated by synaptically-released glutamate

**Figure 4:**
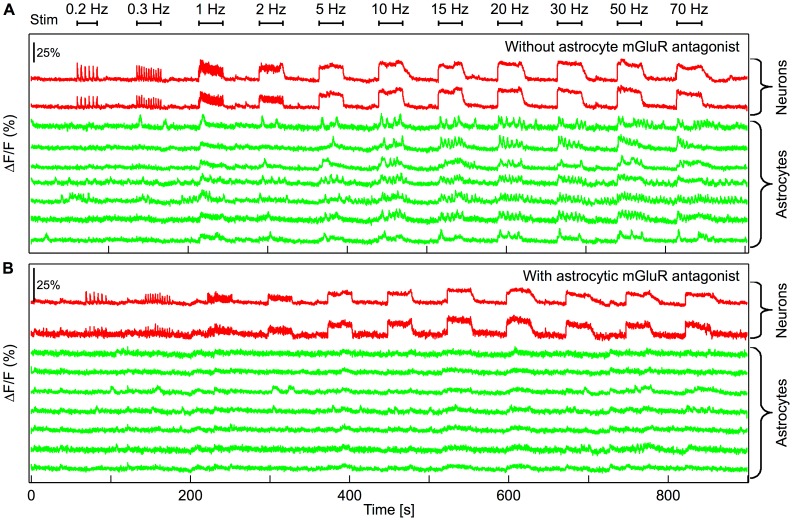
Astrocytic response to neuronal activity in the presence of neuronal AMPAR and NMDAR/kainate antagonists, and astrocytic mGluR1 and mGluR5 antagonists. **A**, Ca^2+^ traces of two selected neurons (in *red*), showing stimulated activity according to a multi-frequency protocol, and seven selected astrocytes (in *green*) in presence of neuronal AMPAR and NMDAR/kainate antagonists. **B**, Representative traces of same cells and stimulation protocol as in A, showing no [Ca^2+^]_i_ elevations in the presence of neuronal AMPAR and NMDAR/kainate antagonists, and astrocytic mGluR1 and mGluR5 antagonists.

We next explored astrocytic activation in response to neuronal activity. To maximize neuronal activation we used nine stimulating electrodes and applied a stimulation protocol consisting of 30 s current pulse trains at frequencies ranging from 0.2 to 70 Hz. At low stimulation frequencies (<1 Hz), single spike patterns are apparent in neuronal Ca^2+^ traces (Fig. 4A, *red* traces). For higher frequencies however, the neuronal response saturates due to slow dynamics and high affinity of the Ca^2+^ dye.

Fig. 4A also displays the Ca^2+^ responses of selected astrocytes (*green* curves). Strong changes in astrocytic Ca^2+^ are observed in response to stimulated neuronal activity. The central result in this work is that the astrocytic responses are highly dependent on neuronal activity frequency: globally, astrocyte tends to respond only when the stimulation frequency becomes large enough. A similar effect was observed in 9 experiments, from 7 different cultures. It should be noted that, whatever the stimulation frequency, the responsive astrocytes were uniformly distributed around the stimulating electrode. Since the astrocyte response does not depend on the distance to the electrode, the possibility of direct electrical stimulation, from the electrode to the astrocyte, is unlikely (S4C-S4E Fig.). To further dismiss the hypothesis that astrocytic response is due to direct electrical stimulation, rather than a result of stimulation-triggered neuronal activity, we conducted control experiments where sodium channel blockers (TTX) were applied to the cultures. Application of TTX eliminated neuronal activity, as expected, but also abolished the nontrivial astrocytic activity (S5 Fig.). Such a disappearance of astrocyte response in the presence of TTX strengthens the notion that astrocytes are not directly activated by the electrodes but by the neuronal activity resulting from electrode stimulation.

A candidate molecule to support the above-described neuron-astrocyte communication is glutamate [5], [46]–[50]. To explore the role of glutamate as the biological transmitter underlying the neuron-astrocyte activity, we applied mGluR1 and mGluR5 antagonists (LY367385 and MPEP respectively). Fig. 4B shows that in those conditions, the neuronal Ca^2+^ traces are essentially similar and faithfully follows electrical stimulation. However astrocytic activity is completely abolished as a result of blocking mGluRs. These experiments yield two important conclusions. First, in our experimental conditions, calcium dynamics in the neuron somata does not appear to be significantly dependent on mGluR group I receptors. More importantly, these experiments indicate that the neuron-astrocyte communication evidenced here is mediated by glutamate activation of astrocytic mGluR group I receptors.

### The astrocyte Ca^2+^ response depends on the frequency of neuronal stimulation

**Figure 5:**
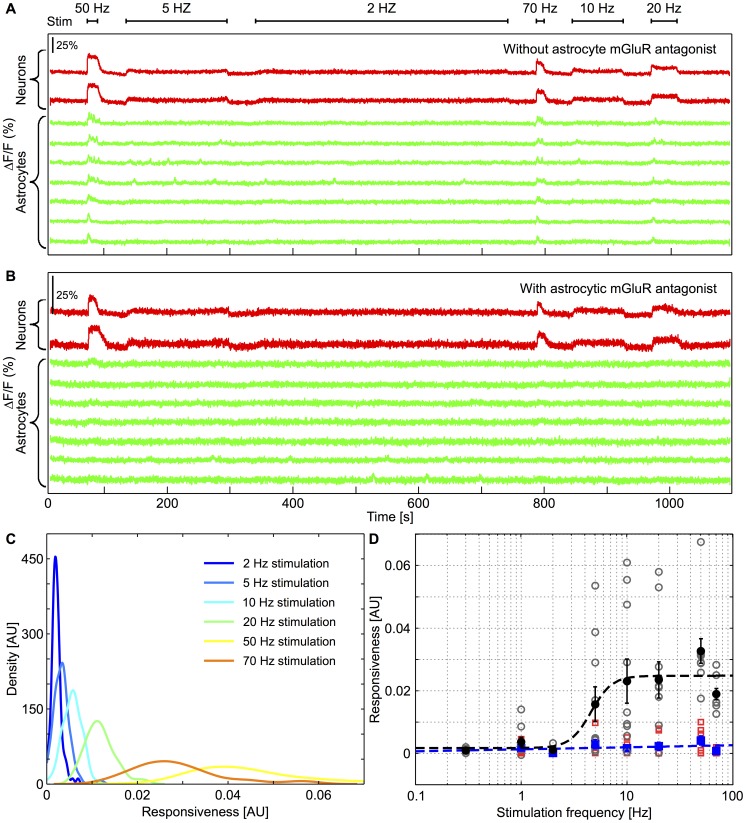
Frequency dependent astrocytic response to neuronal activity. **A**, Ca^2+^ traces of two selected neurons (in *red*), showing stimulated activity according to a random multi-frequency protocol, and seven selected astrocytes (in *green*) showing frequency dependent [Ca^2+^]_i_ elevations in response to neuronal activity. Experiments were performed in the presence of neuronal AMPAR and NMDAR/kainate antagonists. **B**, Ca^2+^ traces of same cells and stimulation protocol as in A, showing no astrocytic [Ca^2+^]_i_ elevations in the presence of neuronal AMPAR and NMDAR/kainate antagonists, and astrocytic mGluR1 and mGluR5 antagonists. For each electrical stimulation frequency, single-cell astrocytes responsivenesses were very variable. Their distribution for the experiment displayed in A is shown in **C**, Increasing the stimulation frequency leads to increases in average astrocyte responsivenesses but it also increased responsiveness variability. **D**, Astrocytic population responsiveness versus stimulation frequency. *Grey* empty circles are population responsivenesses for experiments performed with NMDAR & AMPAR but without (astrocytic) mGluR antagonists (*n* = 284). Corresponding mean result, standard errors (*black* circles and bars), and sigmoid fit (*black dashed* line) are also illustrated. *Turquoise* empty squares are population responsivenesses obtained in the presence of both NMDAR & AMPAR and mGluR antagonists (*n* = 239). Corresponding mean results and standard errors and linear fit are shown as *blue* squares (with bars and *dashed blue* line, respectively).

To further test the above indication that stimulation frequency is indeed the significant parameter affecting astrocyte response, we applied an alternative stimulation protocol, in which we varied the frequency of the electrical stimulation but kept the number of stimulations constant (the stimulation duration was thus inversely related to its frequency). In addition, the order with which the stimulation frequencies were applied to the MEA was chosen at random, so as to avoid artifactual responses such as cell fatigue or dye poisoning. The goal of this alternative stimulation protocol was to distinguish an astrocyte response that would depend on the stimulation frequency from a response that depends on the number of neuronal spikes in an accumulative manner. Fig. 5A shows that astrocytic calcium activity in response to neuronal stimulation is indeed frequency dependent since in response to this alternative protocol, the astrocyte still tend to respond only to the largest stimulation frequencies. Furthermore, as with the stimulation protocol of Fig. 4, the application of mGluR1 and mGluR5 antagonists abolished astrocyte calcium activity in response to neuronal activation (Fig. 5B).

To quantify the above results, we computed the single-cell responsiveness of an astrocyte as the increase of the calcium signal of this astrocyte that is specifically triggered by the stimulation (see Methods). Fig. 5C displays the distributions of the single-cell responsivenesses of all the activated astrocytes in the experiment of Fig. 5A. For low stimulation frequencies, the single-cell responsivenesses are essentially peaked around a very low mean value. When the stimulation frequency increases, the distributions of single-cell responsivenesses get much broader, thus revealing increasing cell-to-cell variations in the response, but the average value of the distribution increases rapidly. Fig. 5D shows the evolution of the average value of the single-cell responsivenesses distribution (referred to as the *population average*, see *Methods*) as a function of the stimulation frequency (each data point on the figure shows the population average of a single experiment). In control conditions (empty *grey* circles), the population responsiveness is very low below a frequency threshold and increases rapidly above this threshold. This sigmoid-like response thus defines an onset frequency that varies from one astrocyte to the other in an experiment and between experiments, as a result of network and intrinsic astrocyte parameters. The variability of the onset frequency will be further explored below using a computer model. The average responsiveness over the experiences (black circles in Fig. 5D, total of *n* = 284 cells) displays a sigmoid shape (dashed line) with an onset frequency of 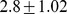 Hz (95% confidence level). Fig. 5D also shows the population responsiveness in the presence of mGluR group I antagonists (*n* = 239) (*empty red squares*). In agreement with the traces in panel B, blocking mGluR receptors suppresses population responsiveness throughout the frequency range. Linear fit of the average population responsiveness (*full blue squares*) yields a roughly zero slope (*dashed blue line*) thus confirming the absence of astrocyte response over the whole frequency range. Taken together, these results show that astrocytic activation in response to neuronal activity is nonlinear with a sharp frequency onset and mediated by glutamate and mGluR type I receptors.

### Spectral analysis of astrocytic [Ca^2+^]_i_ dynamics

**Figure 6:**
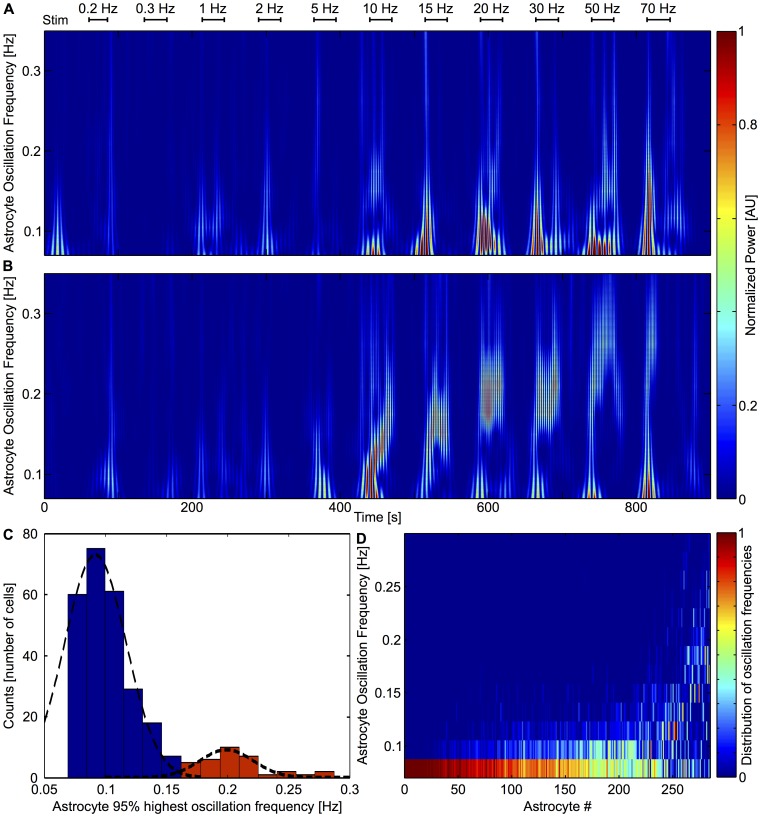
Spectral analysis of astrocytic [Ca^2+^]_i_ oscillation with wavelet analysis. **A**, Time frequency analysis of a representative astrocyte showing typical low frequency oscillation (Type I). **B**, Time frequency analysis of a representative astrocyte showing higher frequency response that increases with stimulation frequency (Type II). **C**, Histogram of the astrocyte maximal frequency (see *Methods*). Typical patterns are colored and fit by a Gaussian distribution (Type I cells in *blue*, and Type II cells in *red*). **D**, Distribution of astrocyte oscillation frequencies. Each column corresponds to one astrocyte and shows its oscillation frequency spectrum (binned at 0.02 Hz). Astrocytes were ranked according to their mean oscillating frequency; most of them oscillate at low frequencies but around one third (right part of the panel) responded to stimulations with oscillations as high as 0.2 Hz. (*n* = 284 cells).

To further characterize astrocytic dynamics in response to neuronal activity we investigated the [Ca^2+^]_i_ oscillation spectrum using time-frequency analysis with Wavelet transforms (Fig. 6). In our protocol, stimulations are given every 30 s. This 0.033 Hz frequency will of course be present in the spectrum of the time series for background and astrocytic activity. Therefore, to derive the astrocytic spectral patterns we set a cutoff frequency of 0.07 Hz so as to get rid of the 0.033 Hz stimulation. As shown in this figure, the time-frequency analysis evidences two types of astrocyte responses. In the first type of response (Type I, Fig. 6A), the oscillation frequency does not change much after the onset frequency and persistently displays low frequency oscillations at 0.09–0.16 Hz whatever the stimulation frequency. The other type of astrocyte response (Type II, Fig. 6B) globally shows larger oscillation frequencies (within 0.16–0.22 Hz) and a marked tendency to increase its maximal response frequency when the stimulation frequency increases. Rather than corresponding to two very distinct types of astrocytes, these two types of response should be considered as the two extreme ends of a spectrum of responses. While the experimental noise prevent us from asserting whether type I responses might display a small increase in oscillation frequency when the stimulation frequency increases, the reader should keep in mind that the main distinction between these two types of responses is their maximal oscillation frequencies. Accordingly, Fig. 6C shows an histogram of the maximal oscillation frequency of all the astrocytes recorded in control conditions and for all stimulation frequencies (as described in methods section). This bimodal histogram confirms the existence of the two distinct response types, with type I response peaking at a maximal frequency around 0.1 Hz and type II maximal response centered around 0.2 Hz. Additionally, Fig. 6D shows a concatenation of the spectra (*y*-axis) of all measured astrocyte responses, after ranking of the cells by increasing value of the mean oscillation frequency (*x*-axis). This confirms that the response of a given astrocyte is either close to type I (with a spectrum essentially centered around 0.1 Hz) or to type II (with a much broader spectrum, ranging from 0.1 to 0.2 Hz), but does not change during the time of the experiment (note that the spectra shown here take into account whole experiment, i.e. the whole range of stimulation frequencies).

Therefore, our spectral analysis of the astrocyte calcium response to neuronal activation indicates the existence of two types of astrocytic responses: above the onset stimulation frequency, type I astrocyte responses display low oscillation frequencies that are seemingly independent of the stimulation frequency, whereas type II astrocyte responses display high oscillation frequencies and increased oscillation frequencies as the stimulation frequencies increase.

### A computer model of astrocyte activity in cultures

To improve our understanding of the biophysical mechanisms at play in the experimental observations reported above, we developed a realistic computer model of neuron-to-astrocyte glutamatergic signaling, that could reproduce the essential features of the astrocyte Ca^2+^ response to neuronal activity observed in experiments. In this computer model, the spatial location of the astrocytes and neurons, the identities of the astrocytes that are directly stimulated by the neurons as well as the gap-junction channel (GJC) couplings between astrocytes were all inferred from the MEA experiments described above (see Methods), so each computer simulation corresponds in one-to-one manner to the experiment from which its parameters were deduced. This one-to-one correspondence allowed us to capture part of the experiment-to-experiment variability without the need to estimate individual intracellular parameters.

Fig. 2A shows the location of cell bodies in a typical MEA experiment, with astrocytes cell bodies segmented in green contours. Starting from this image, we use Voronoi tessellation to define the anatomical domain of each astrocyte, as shown in Fig. 2B with the model astrocytes as green circles. Since astrocyte coupling via GJC is expected to be restricted to the boundary between the astrocyte anatomical domains, every pair of astrocytes that shares an anatomical domain boundary was coupled via GJC in the computer model (black links). Boundaries between anatomical domains are best illustrated on Fig. 2C in which anatomical domains where constructed with Voronoi tessellation (*light gray* lines). On this example, an astrocyte A shares its domain boundaries (*thick blue* lines) with 7 other astrocytes (B1 through B7). If all these astrocytes are correctly characterized by their Ca^2+^ activity (i.e. if they are not classified as unresponsive cells), A will be connected to its 7 neighbors in the reconstructed network. Finally, those astrocytes that displayed a calcium response that was strongly time-locked to the electrical stimulations in the experiments were stimulated by glutamate release in the computer model (yellow lightnings on Fig. 2B). Fig. 2D display the statistics of the astrocyte networks obtained by this procedure (all the experiments were sampled). The coupling degree of each astrocyte, i.e. the number of astrocytes it is GJC coupled to, is typically small, with roughly 1/5^th^ of the cells non connected and 1/3rd of the cells coupled to a single other astrocyte (Fig. 2D). Most of the connected astrocyte clusters comprise a small number of astrocytes (1 to 3, Fig. 2D inset) but very large clusters, where more than 60 astrocytes are interconnected, also occur, albeit with small probability (Fig. 2D, inset, stars). The number of isolated astrocytes (≈80) is comparable to the number of astrocytes that are part of a cluster larger than 10 astrocytes (≈110). These statistics substantially differ from the reported size of astrocyte networks in intact tissue (up to hundreds of astrocytes in the neocortex [51]). This discrepancy can be accounted for by two main phenomenon: (1) astrocytes that do not exhibit Ca^2+^ activity are absent from our reconstructed networks, thus decreasing the overall connectivity; (2) *in vitro* culture imposes a mostly 2D embedding of astrocytes, which further reduces the number of neighbors that they can contact.

**Figure 7:**
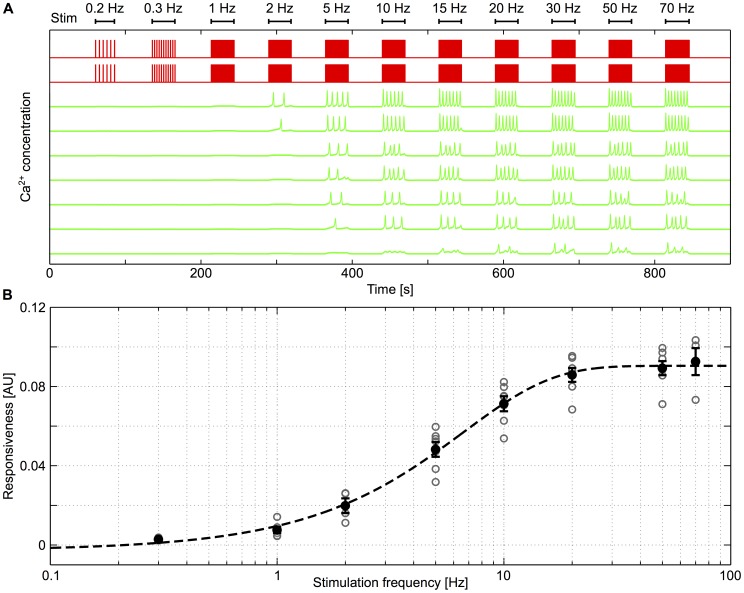
Model astrocytes also respond in a frequency-dependent manner to neuronal stimulation. **A**, Astrocyte calcium signals (*green* traces) show a variety of responses, as in the experiments. Some of them start responding at frequencies as low as 2 Hz (top trace) while others need up to 20 Hz to elicit a significant response (bottom trace). Astrocytes were stimulated using the *red* neuronal spike train, stimulation frequencies are indicated on top of the figure. **B**, Using the same method as in the experiments (Fig. 3C), population responsiveness was computed in the simulations and plotted as a function of stimulation frequency for all modeled cultures. *Grey* circles denote population responsivenesses and black dots their average value. Error bars indicate standard error and the *black dashed* line shows the sigmoidal fit characterizing the frequency-dependent astrocyte response (n = 130 cells).

Fig. 7A illustrates the simulation results corresponding to the experiment reported in Fig. 4A above, with similar graphical conventions. The simulated astrocytic Ca^2+^ traces are shown in *green* and the synaptic stimulations at different frequencies as *red* traces. As the stimulation frequency increases, simulated astrocytes start responding by [Ca^2+^]_i_ oscillations. However, just like in the experimental traces, the onset frequency varies from cell-to-cell. This behavior as well as the overall aspect of the simulated Ca^2+^ traces is in agreement with the corresponding experimental observations (see e.g. Fig. 4A). The biophysical consistency of our model is also supported by the evolution of the population responsivenesses based on the simulated Ca^2+^ traces (Fig. 7B). In close analogy with the experiments (see Fig. 5D), the average population responsiveness is a sigmoid function of the frequency of stimulation and is characterized by a marked onset of 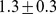 Hz (95% confidence level) (*n* = 130), consistent with experimental data (Fig. 5D).

**Figure 8:**
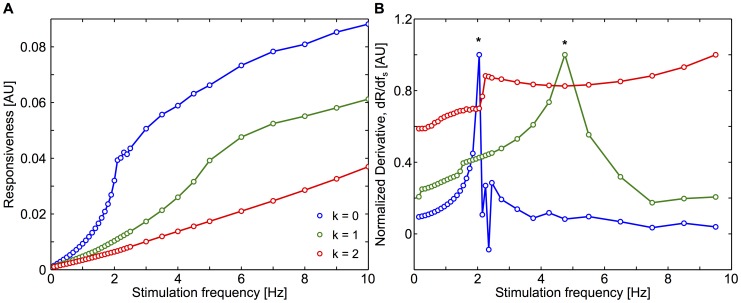
Model astrocytes display a coupling degree-dependent onset. **A**, Single-cell astrocyte responsiveness as a function of stimulation frequency and for different coupling degrees (i.e. number of unstimulated astrocytes to which the stimulated one is coupled). **B**, Normalized derivatives ((*dR*/*df*_s_)/max(*dR*/*df*_s_)) of the curves in A; peak values are denoted by stars (exact onset frequencies). Isolated astrocytes (*k* = 0, *blue* line) display a fast increase in responsiveness just before 2 Hz while astrocytes which are linked to one astrocyte (*k*  = 1, *green* line) have a much later onset, around 5 Hz. For *k*  = 2, *red* line the exact onset frequency is above 10 Hz.

One virtue of our computer model is that the single-cell responsiveness in the model can be characterized using a number of stimulation frequencies that is much larger than in the experiments. Fig. 8A shows the single-cell responsiveness of three model astrocytes that are GJC coupled to zero, one or two unstimulated astrocytes, respectively. This linear-linear representation confirms the existence of a threshold frequency (exact onset frequency, see the methods section) for the responsiveness. The inflection point in these curves corresponds to the stimulation frequency at which the first Ca^2+^ oscillation appears. For lower stimulation frequencies, Ca^2+^ levels slowly increase in response to increases in stimulation frequency but do not display oscillations. This sub-threshold regime could not be seen in the experimental data, possibly because of the relatively high experimental noise. In addition, the figure shows that the exact onset frequency increases with the coupling degree of the astrocyte. The existence of the exact onset frequency can be more firmly established by the presence of an inflection point in the curves. Fig. 8B shows the (numerical) derivative of the curves of Fig. 8A. For the isolated astrocyte (*k* = 0, *blue* curve), a clear inflection point is found at around 2 Hz (i.e. close to the onset frequency reported for the population responsivenesses in the experiments, see Fig. 5D), whereas it is close to 5 Hz in the singly-connected case (*green* curve) and larger than 10 Hz for *k* = 2 (*red*). Therefore, the model not only confirms the existence of an onset stimulation frequency for the responsiveness, it also predicts that this onset frequency increases with the astrocyte coupling degree.

### A biophysical explanation for the onset frequency of astrocyte responsiveness

Our analysis hitherto supports the notion that astrocyte responsiveness to neuronal stimulation is a nonlinear and sigmoid function of the neuronal stimulation frequency, thus describing an onset frequency. Here, we use our computer model to propose a mechanism supporting this nonlinear response. In the model, this threshold frequency is mostly due to the Ca^2+^-Induced- Ca^2+^-Release (CICR) process, that underlies most of the Ca^2+^ dynamics in astrocytes [52]. CICR is a self-amplifying mechanism by which Ca^2+^ influx from the endoplasmic reticulum (ER) to the cytoplasm is activated by cytosolic Ca^2+^ itself. But CICR can only be triggered if intracellular IP_3_ reaches a threshold concentration [53]. In turn, this IP_3_ threshold mainly depends on the binding affinity of the IP3R/Ca^2+^ channels.

**Figure 9:**
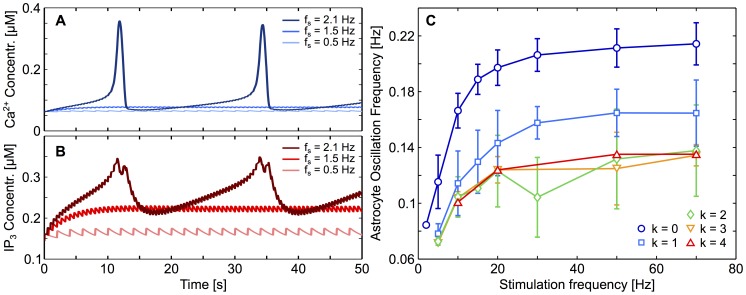
Network parameters affect astrocytic [Ca^2+^]_i_ traces. **A**, Ca^2+^ and **B**, IP_3_ traces of isolated astrocytes in response to different stimulation frequencies. Above 2 Hz, as shown on Fig. 7, astrocytes start responding with Ca^2+^ oscillations. Below 2 Hz, IP_3_ and Ca^2+^ levels reach a frequency-dependent steady-state; when these steady-state concentrations are high enough to trigger CICR (i.e. for high enough stimulation frequency), astrocytes respond with large Ca^2+^ oscillations. **C**, Increasing the stimulation frequency increases the astrocyte oscillation frequencies. Whatever the coupling degree *k*, the oscillation frequency reaches a plateau for high stimulation frequencies. The height of this plateau however strongly depends on the astrocyte coupling degree. Isolated astrocytes oscillate much faster than connected ones. Error bars denote standard deviation (n = 130 astrocytes).

This property is illustrated in Fig. 9AB, which shows the simulated Ca^2+^ and IP_3_ traces of a model (non-connected) astrocyte in response to neuronal stimulation. According to Fig. 8, in such a non-connected astrocyte, the onset frequency is around 2 Hz. For subthreshold stimulation frequencies (e.g. 0.5 or 1.5 Hz), the intracellular IP_3_ concentration first slowly increases upon each stimulation pulse then stabilizes around a stationary value (Fig. 9B, *red* trace). However since this stationary value is below the threshold IP_3_ concentration for CICR, the resulting levels of intracellular Ca^2+^ slightly increase to stabilize to a steady value and no effective response of the astrocyte to stimulation is observed (Fig. 9A). The scenario changes radically when the stimulation frequency is above the onset frequency (e.g. 2.1 Hz in Fig. 9AB). In this case the intracellular IP_3_ concentration crosses the CICR threshold at roughly *t* = 10 seconds, which yields a characteristic IP_3_ peak and, as a result of the self-amplification of the CICR, a large Ca^2+^ response (Fig. 9A).

Examination of the dynamics of the underlying signaling pathway in the simulations (S6 Fig.) reveals that this IP_3_ peak is mostly due to a sharp increase of production by Ca^2+^-activated PLCδ, that is itself provoked by the early increase of Ca^2+^ at the onset of CICR. Initiation of the IP_3_ peak further amplifies the whole process by a fast increase of IP3R channels open probability. However, since part of IP_3_ degradation process is also Ca^2+^-dependent, the IP_3_ degradation rate parallels the steep Ca^2+^ rise. As a result, excess IP_3_ is readily degraded as soon as Ca^2+^- reaches high enough values and the IP_3_ surge recesses back. Finally, CICR itself switches off due to the Ca^2+^-dependent inactivation of IP3Rs.

Taken together, our model indicates that the existence of the onset frequency would be due to a supralinear response of intracellular IP_3_ that triggers the CICR system only when the stimulation frequency is large enough.

### GJC coupling may account for the different frequency response types

**Figure 10:**
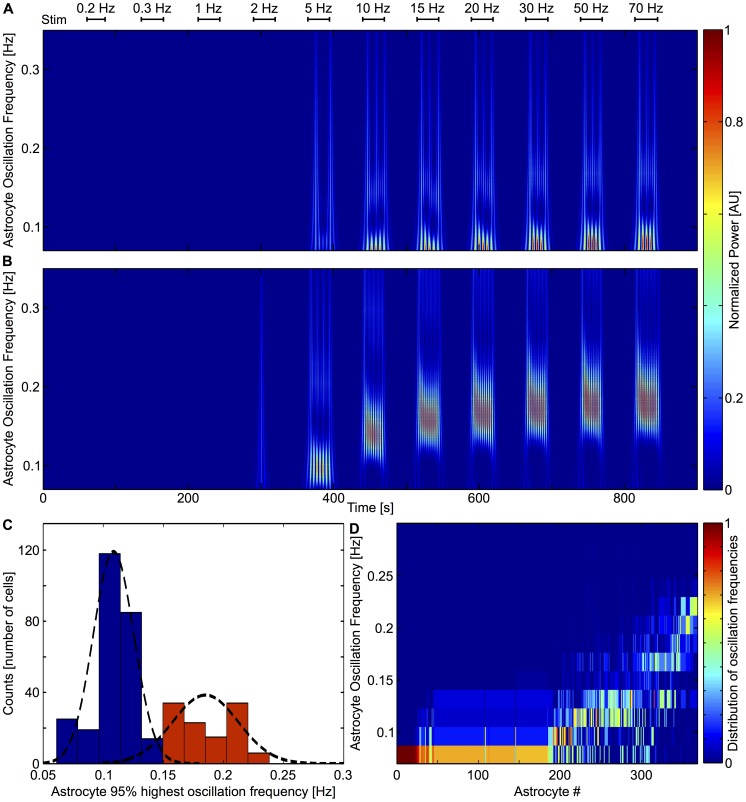
Spectral analysis of model astrocytic [Ca^2+^]_i_ oscillations by wavelet analysis. **A**, Time-frequency analysis of a representative astrocyte showing typical low frequency oscillation (Type I). **B**, Time-frequency analysis of a representative astrocyte showing typical high increasing frequency oscillation (Type II). **C**, Histogram of the maximal oscillation of astrocytic populations. Typical patterns are colored and fitted by a Gaussian distribution (Type I cells in *blue*, and Type II cells in *red*, total *n* = 373 cells). **D**, the distribution of astrocyte oscillation frequencies. Each column corresponds to one astrocyte and contains its oscillation spectrum (binned at 0.02 Hz). Astrocytes are ranked according to their mean oscillating frequency; most oscillate at low frequencies but around one third (right part of the panel) respond to stimulations with frequencies as high as 0.2 Hz, thus matching the experiments (cf. Fig. 4D).

In the above experiments, astrocyte Ca^2+^ oscillation frequencies show large cell-to-cell variability, up to a two-fold change across different cells. Our model astrocytes also display such variations in oscillation frequencies as shown on Fig. 7A (the two astrocytes displayed at the top of the panel oscillate much faster than the 5 ones displayed at the bottom). To further analyze our simulation results, we investigated their spectral dynamics, applying the same time-frequency analysis as we did for our experimental results (Fig. 10). In remarkable agreement with the analysis of our experimental results (Fig. 6), our simulations displayed two oscillation patterns: Type I oscillations are characterized by nearly constant low frequency oscillations around 0.1 Hz mostly independent of the stimulation frequency whereas Type II oscillations are higher (0.1–0.2 Hz), with frequencies that increase when the stimulation frequency grows (Fig. 10AB). Although exact quantitative match is not to be expected, our computer model thus qualitatively matches experiment observation, including the two response types (Type I and Type II). We must stress here that these two response types should not be considered as two distinct astrocyte types but rather as two opposite ends of a spectrum of astrocytic responses. The good match with experimental results is further illustrated by the comparison of the maximal frequency histogram (Fig. 10C) and full frequency distributions (Fig. 10D) of the simulated astrocytes (compare to Fig. 6C and D). Just like in the experimental data, in our computer simulations, astrocytes are either close to Type I or Type II but do not switch between the two response types during the simulation (Fig. 10D).

According to our model this intrinsic characteristic can be explained on the sole basis of the GJC coupling between astrocytes. Fig. 9C shows how the mean oscillation frequency varies with the stimulation frequency depending on the effective coupling degree of the stimulated astrocyte (i.e. the number of unstimulated astrocytes it is GJC coupled to). When connected to two unstimulated astrocytes or more, the amplitude of the increase of the astrocyte response frequency with the neural stimulation frequency is very moderate. The response frequency is clipped between 0.08 and 0.14 Hz, whatever the stimulation frequency. These characteristics correspond to the properties of Type I astrocyte responses observed both in vitro (Fig. 6A) and in the simulations (Fig. 10A). In contrast to the data obtained from the simulations of the model, the moderate rise of the oscillation frequency in the experimental data is not visible, most certainly because of measurement noise. The low values of the oscillation frequencies for type I responses are however clearly observed in the experimental data and are well reproduced by the model. On the other hand, when the simulated astrocyte is coupled to a single unstimulated astrocyte, or even when it is not coupled at all, the amplitude of the increase of the astrocyte response with increasing neuronal stimulation is larger (Fig. 9C): starting from 0.08 Hz (for frequency close to around the onset frequency), it sharply increases with the stimulation frequency, until it reaches oscillation frequencies above 0.2 Hz, thus a roughly three-fold increase compared to the onset frequency. These properties match well the Type II astrocyte responses of Fig. 6B and 10B. Therefore, our simulations suggest that the two types of astrocyte responses identified above actually correspond to astrocytes with different coupling degrees: Type I response would correspond to highly connected astrocytes (coupled to more than 2 unstimulated astrocytes) whereas Type II would be the response of isolated (or weakly coupled) astrocytes. This phenomenon can be explained by considering unstimulated coupled astrocytes as IP_3_ sinks which hinders its local accumulation, inducing slower oscillations (see [37] for further detail on the influence of network topology).

### Single-cell onset frequency correlates with the oscillation response frequency

**Figure 11:**
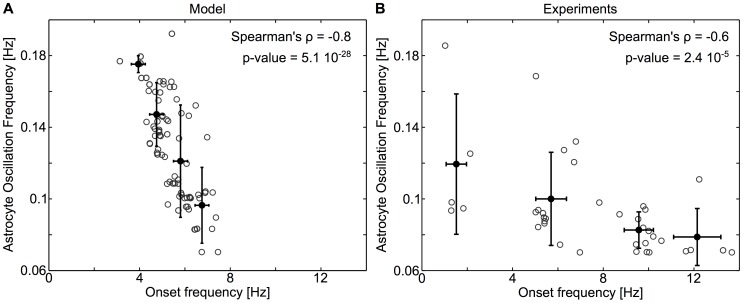
The oscillation frequency is significantly and negatively correlated to the onset frequency. **A**, In the model, astrocytes that display high frequency oscillations also respond earlier (for smaller onset frequencies) than slowly oscillating astrocytes. The analysis was restricted to stimulated astrocytes (*n* = 130). **B**, The same effect is visible in experimental data; while the range of onset frequencies wider, the negative correlation between oscillation frequency and onset frequency is very significant. The analysis was restricted to strongly stimulated astrocytes, which responded to at least one stimulation in less than 1.5 s (*n* = 40). For A and B, *grey* circles denote single-cell astrocyte responses from all simulations or cultures, while *black* dots denote averages of the data after splitting in 4 classes. Error bars show corresponding standard deviation. Astrocytes were submitted to 10 Hz stimulations and onset values were computed as explained in the methods section.

Fig. 9C also gives a hint about the relation between the onset stimulation frequency and the oscillation response of the stimulated astrocyte. In those curves, the first data point (for each coupling degree) gives an indication of the onset frequency. Therefore, one can see from the figure that the larger the effective cell coupling degree, the larger the onset frequency. We have further tested this hypothesis in Fig. 11. For every stimulated astrocyte simulated above, we plotted its average oscillation frequency (for neuronal stimulations at 10 Hz) versus its single-cell onset stimulation frequency (*grey* empty circles, Fig. 11A). This figure shows a very significant negative correlation: the larger the onset stimulation frequency, the smaller the calcium oscillation frequency.

A major interest of this prediction is that it can easily be tested in the experimental data since it does not necessitate to measure the astrocyte coupling degree (that we currently cannot measure nor control in our MEA setup). Fig. 11B shows the same plot as Fig. 11A but with the experimental data. Albeit the overall range of onset frequencies exhibited by the astrocytes is much larger than their simulated counterparts, the experimental data confirm that the oscillation frequency of the astrocyte calcium response shows a significant and negative correlation with the onset frequency. These results therefore show that the oscillation frequency of the astrocytes is not independent of their onset frequency, but both are negatively correlated. As suggested by the model, the primary cause of this negative correlation is suspected to be the coupling degree of the stimulated astrocyte, with onset frequencies that increase and response frequencies that decrease with astrocyte coupling degree.

## Discussion

The activation of intracellular calcium signaling in astrocytes by neural activity has been amply documented during the last twenty years. It is even at the origin of the recent renew of interest for astrocytes and neuron-glia interactions [54]. However, how the temporal pattern of astrocyte calcium response varies with the properties of the neuronal stimulation is still poorly understood. Here, we provide unequivocal evidence that the astrocyte calcium response depends on the neuronal stimulation frequency in a nonlinear way and features a marked onset frequency (around 2–5 Hz) below which astrocytes do not respond to neuronal stimulation. Moreover, we show that this onset frequency is strongly cell-dependent and can vary by more than a decade (between 1.0 and 13.0 Hz) from one astrocyte to the other (Fig. 11B). Furthermore, we show the existence of two classes of astrocyte response patterns to the neuronal stimulation frequency. Using a realistic biophysical model of glutamate-based intracellular calcium signaling in astrocytes, we suggest that these nonhomogeneous and cell specific response properties may reflect the heterogeneity of astrocyte coupling and, more specifically, of the astrocyte coupling degree, i.e. the number of distinct astrocytes coupled to the stimulated astrocyte via gap junction channels.

The notion that the calcium response of astrocytes is not elicited if the stimulation is too weak is often encountered in the astrocyte literature. In vivo, when the astrocyte response is triggered by a physiological stimulus (image, whisker stimulation, odor stimulation, and even motor behavior), a minimal stimulation amplitude or frequency is usually needed to elicit a response by the astrocyte [49], [50], [55], [56]. A recent in vivo study even relates neuronal activity intensity (elicited by sensory stimulation) and astrocyte responses at a network level in the rat somatosensory cortex [57], showing that subgroups of astrocytes can respond selectively to sensory stimulus. In slices, the calcium response of astrocytes is generally triggered by intense and high-frequency stimulations of neuronal afferents (stimulation trains at 20–50 Hz) [48], [58] while weaker and low frequency stimulations (single pulses at 0.1–10 Hz) usually fail to induce a calcium response in astrocyte cell bodies [5], [47]. Our results push these results further since they locate a precise onset frequency and they demonstrate that the astrocyte response is indeed dependent on the stimulation frequency, excluding a potential implication of the number of applied stimulations. Although based on cell culture, which is often and righteously criticized for its uncertain correlation with in vivo conditions, our MEA approach presents decisive assets to quantify astrocyte response to neuronal stimulation: it allows to quantify calcium astrocyte dynamics at the single-cell level and control astrocyte stimulation by neurons. We could moreover test many different stimulation frequencies on the same cells and benefit from complete control of the electrical stimulation frequency, duration and sequence. In particular, we could vary the stimulation frequency while keeping the number of stimulations constant and the order of stimulation frequency random (Fig. 5). This experiment provided the requisite arguments to exclude a potential dependence on the number of stimulations, fatigue or toxicity effects.

An additional concern is the possibility that the physiological state of astrocytes in culture may not really correspond to physiological in vivo conditions but be closer to reactive astrogliosis, a graded process that occur in response to pathological states. Resulting effects on astrocytes can range from small changes in gene expression to dramatic changes in cellular morphology and even to the formation of glial scars [59], [60]. We did not observe obvious signs of astrocytic hypertrophy nor excessive expression of GFAP protein, which are signs of reactive astrogliosis [59], [61], [62]. However, since we assessed both of these characteristics by visual inspection of immunostaining pictures of cultures (as displayed on Fig. 2C and S5B Fig.), we cannot definitively rule out the possibility of mild reactive astrogliosis. Cultured astrocytes are notoriously different from adult astrocytes in vivo in both their morphology and their gene expression profiles. In particular, cultured neonatal astrocytes obtained with the McCarthy-DeVellis method [63] are known to display markers specific to reactive astrogliosis [61]. It is however not clear whether this effect also accompanies different culture methods. McCarthy-DeVellis astrocytes are purified astrocyte-only cultures whereas our cultures, in addition to astrocytes, also include neurons (which are known to regulate gene expression in astrocytes [64]) and other glial cells, thus possibly changing the way astrocytes react to cell culture. Altogether, since we could not conclusively assess whether astrocytes in our cultures are reactive or not, our results should be interpreted with care and might reflect non-physiological behavior. In particular, reactive astrocytes are known to display altered Ca^2+^ signaling during pathological states such as ischemia [65], Alzheimer's disease [66] and brain trauma [67]. Of particular relevance to our study, reactive astrogliosis has also been shown to mediate both G-protein coupled receptor signaling and increases in Cx43 expression [68]. Therefore, our results could thus prove pertinent to the study of the implication of astrocytes in brain pathologies.

A dimension that is not accounted for by our study is the multi-scale characteristics of calcium signaling in astrocytes. Indeed, our current imaging methods restrict our observations to the calcium dynamics taking place in the astrocyte cell body. In addition to the cell body, the astrocyte cell extends complex shaped processes that also host intense calcium signaling. It is increasingly recognized that astrocytes also respond to low frequency synaptic activity with calcium elevations that are restricted to their processes [18], [69]. In physiological conditions, the frequency of these subcellular responses in astrocyte processes is higher than the frequency of somatic Ca^2+^ increases. While most events are restricted to small portions of processes [69], [70], some of them do propagate to the astrocyte soma [71] even in vivo [70]. Astrocytes could thus be seen as integrators of neuronal activity; in the present study, we only considered the integration at the somatic level, which can be seen as the “readout” of a much more complex subcellular signaling. While the link between subcellular processes activity and somatic activity remains to be addressed [71], an exciting possibility would be that the structural complexity generated by the astrocytic processes could help explain the frequency-dependent response that we observe here. In particular, future experimental approaches should address whether the frequency-dependent response also holds at a subcellular level. The study of subcellular calcium transient in astrocyte process is a recent development based on advanced technologies [18], [69] that will likely yield significant insights on astrocytic calcium signaling in the near future.

Using our computer model, we aimed at validating the hypothesis that the observed cell-to-cell heterogeneity in the astrocyte response could be rooted in the distribution of the coupling degrees: astrocytes coupled to several other astrocytes would need larger neuronal stimulation frequencies to be activated, display smaller oscillation frequencies and their response would be less dependent on the frequency of the neuronal stimulation. The presence of GJC couplings in astrocyte cell cultures is well documented. Connexin 43 (Cx43) and 30 (Cx30) are the two main proteins supporting GJC formation in astrocytes [64], [72], [73]. Both proteins are expressed in astrocytes during both embryonic [74] and postnatal [64], [75] cortical development and their expression is even enhanced in neuron-astrocyte co-cultures such as employed here [64]. The indirect test presented in Fig. 11 (negative correlation between response and stimulation frequencies) supports our predictions. According to the model, high frequency oscillations in astrocytes are associated with disconnected astrocytes. Interestingly, such uncoupled astrocytes have been reported in the mouse barrel cortex [76], in hippocampus and cerebral cortex of the developing mouse brain [77], among olfactory ensheathing cells (non-myelinating glial cells that share some properties with astrocytes) [78], and in cell cultures, where they represent a substantial number of astrocytes (21% in mixed neuron-astrocytes cultures and 44% in astrocyte-only cultures) [79]. To the best of our knowledge, whether these same astrocytes also display faster Ca^2+^ oscillations has not, to our knowledge been conclusively demonstrated. Application of endothelin-1 (ET-1), that reduces GJC coupling, on mixed neuron-astrocyte cultures promoted Ca^2+^ oscillations in astrocytes [80]. Moreover Ca^2+^ oscillation frequency was found to be increased in astrocytes in a pathology that reduces GJC coupling [81]. Both of these effect could however be mediated by other pathways and should not be held as definite proof of the link between coupling degree and oscillating frequency. A more convincing case is provided by [47], [82]; the authors show that nitric oxide gradually increases astrocyte oscillation frequency over periods ranging from 2 to 60 minutes while they specifically state that nitric oxide does not have direct oscillation-inducing effects on astrocytes. Interestingly, although they did not mention it in the articles, nitric oxide is known to reduce GJC coupling between astrocytes [83], [84]. It could thus very well be that the increase in astrocyte oscillation frequency that they witness is directly linked to the reduction of GJC coupling induced by nitric oxide. Unfortunately, Pasti et al. did not test this hypothesis and future experiments will thus be needed to determine whether GJC could be responsible for the observed variability in astrocytic responses. Within our experimental framework, a more direct investigation with gap-junction blockers will provide more biological insight into the observed heterogeneity of astrocyte oscillation frequencies and will be addressed in future explorations. In lieu of the distribution of the coupling degrees, one alternative hypothesis that we could have investigated is that the heterogeneity of the single-cell response could be due to the distribution of the intracellular biochemical parameters (density of mGluR, PLC, IP3R channels, SERCA pumps) that are expected to vary from cell to cell. But here again, this hypothesis would be very difficult to validate experimentally, because it would necessitate to control the cell-to-cell distribution of these parameters. We however plan to explore this path in future works.

## Supporting Information

S1 Fig Illustration of our experimental setup. A combined MEA and calcium imaging setup was used to enable simultaneous neuronal activation through application of electrical stimulation, while recording neuronal and astrocytic cellular activity through Ca^2+^ dynamics.
(TIF)

S2 Fig Safety mapping of electrical activation. **A**. Neuronal Ca^2+^ traces. Top two cells show stable activation, bottom three cells exhibit non-reversible activity. **B**. larger scale of same traces as in A. **C**, Ratio of cells exhibiting irreversible activity as a function of stimulation amplitude, indicating safe zone. **D**, Color coded activation map indicating irreversible activity threshold (bottom three cells in A, B marked by arrows).
(TIF)

S3 Fig Immunostaining to distinguish neurons from astrocytes. **A**, Image of recorded culture with marked cells. **B**, Combined pseudo-color immunostaining image of same field of view and marked cells as shown in A. Red - neuronal marker NeuN. Green – astrocytic marker GFAP. Blue – nuclei visualization agent DAPI. **C**, Spontaneous Ca^2+^ traces of same neurons and astrocytes (in red and green respectively) as marked in A, B. Scale bars are 75 µm. Culture was 14 DIV.
(TIF)

S4 Fig Parameters of neuronal activation. **A**. Ca^2+^ traces of typical stimulated activity. Neurons (in red) respond to electrical stimulation, and astrocytes (in green) exhibit some [Ca^2+^]_i_ elevation. **B**. Spontaneous activity traces of same cells as in A. **C**, Image of recorded culture with specified cells from A, B. Electrode is marked in red, and scale bar indicates 75 µm. Culture was 17 DIV. **D**, Distribution of the neurites of activated cells may be delineated by stimulation triggered averaging. Density of activated neurites is highest in the vicinity of the electrode. **E**, Stimulation threshold as a function of distance from electrode, indicating no clear correlation between distance from electrode and stimulation threshold (standard errors indicated).
(TIF)

S5 Fig Astrocytic response is not a direct effect of electrical stimulation. **A**, Traces of two selected neurons (in red), showing stimulated activity according to protocol, and seven selected astrocytes (in green) in presence of neuronal AMPAR and NMDAR/kainite antagonists. **B**, Traces of same cells and stimulation protocol as in A, showing no neuronal and astrocytic [Ca^2+^]_i_ elevations in the presence of neuronal AMPAR and NMDAR/kainite antagonists, and TTX. **C**, Astrocytic responsivity as a function of stimulation frequency in presence of TTX show no astrocytic frequency dependence (N = 20). Left inset shows image of recorded culture with specified cells from A, B. Electrode is marked in black, and scale bar indicates 75 µm. Right inset is a histogram of responsive astrocytes per area as a function of radius from electrode in µm (N = 277).
(TIF)

S6 Fig Detailed astrocytic response to a 2.1 Hz neuronal stimulation. The spiking behavior displayed by isolated astrocytes for stimulation frequency above 2 Hz (see Fig. 8) can be understood by examining the dynamics of the underlying signaling pathway. **A**, Above 2 Hz, the IP_3_ produced by PLC-β (in orange) leads to small opening of IP_3_R channels, increasing the Ca^2+^ concentration in the cytosol. This increased Ca^2+^ level activates PLC-δ IP_3_ production (in brown). **B**, Detailed view of PLC-β and PLC-δ IP_3_ production during a Ca^2+^ rise. **C**, This positive feedback loop triggers the CICR by increasing the opening probability of IP_3_R channels (in green). Further increases in Ca^2+^ inactivates IP_3_R channels (in red) thus ending the Ca^2+^ rise as it gets reintegrated into the ER. **D**, During this process, IP_3_ is degraded by Ca^2+^-dependent IP_3_-3K enzymes during the Ca^2+^ rise (in purple), and by Ca^2+^-independent IP-5P enzymes (in blue).
(TIF)

S1 Table Model parameters.
(PDF)

S1 Text **Full model description** (Astrocytes dynamics; Synaptic dynamics); **Supporting References.**
(PDF)
