# Supplementary figures and images for: Glutamate Mediated Astrocytic Filtering of Neuronal Activity
Source: PLoS Comput Biol. 2014 Dec 18;10(12):e1003964. doi: 10.1371/journal.pcbi.1003964 (PMC4270452; doi:10.1371/journal.pcbi.1003964)

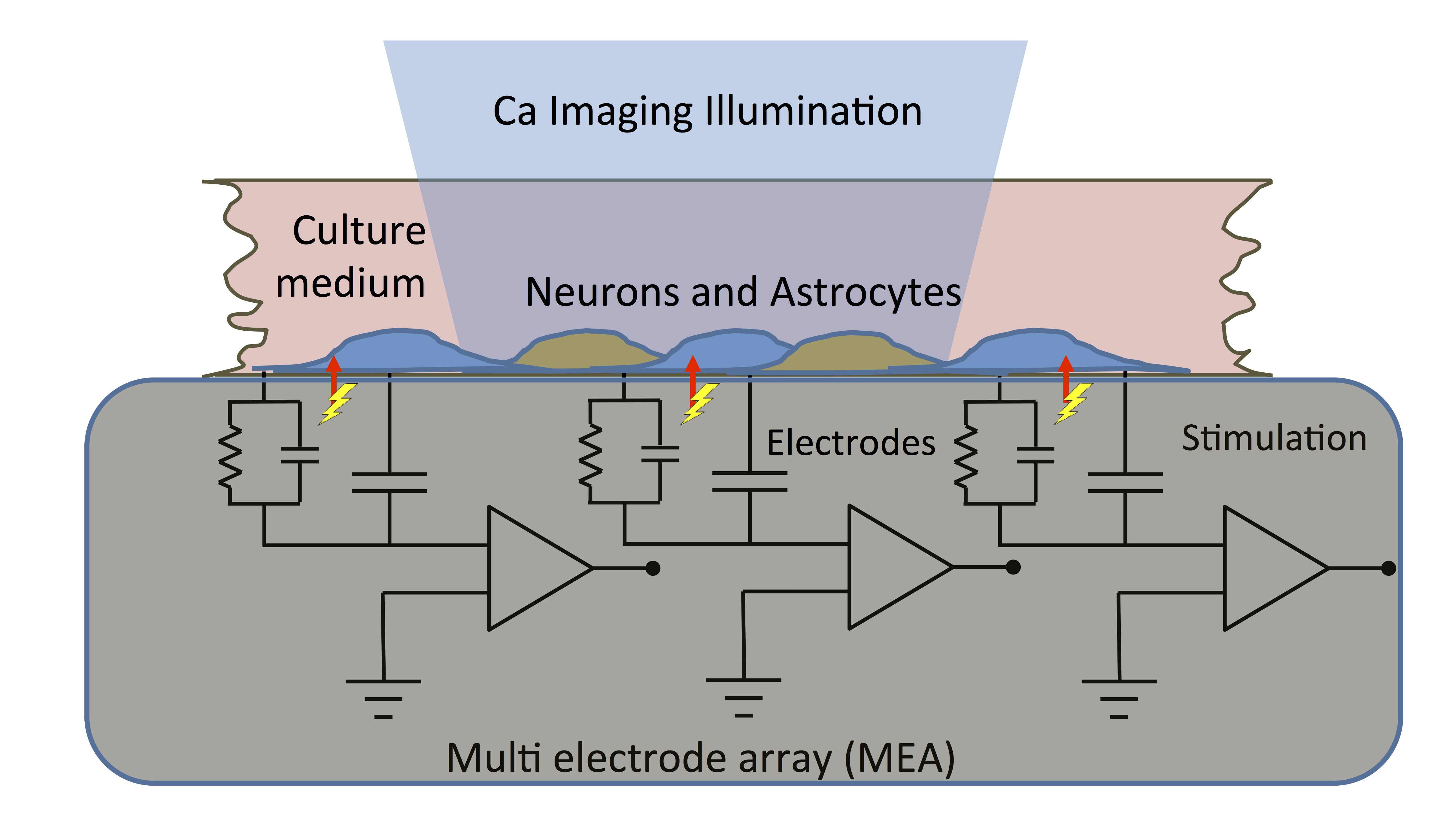

Supplement: S1 Fig — Illustration of our experimental setup. A combined MEA and calcium imaging setup was used to enable simultaneous neuronal activation through application of electrical stimulation, while recording neuronal and astrocytic cellular activity through Ca2+ dynamics. (TIF) [file pcbi.1003964.s001.tif]

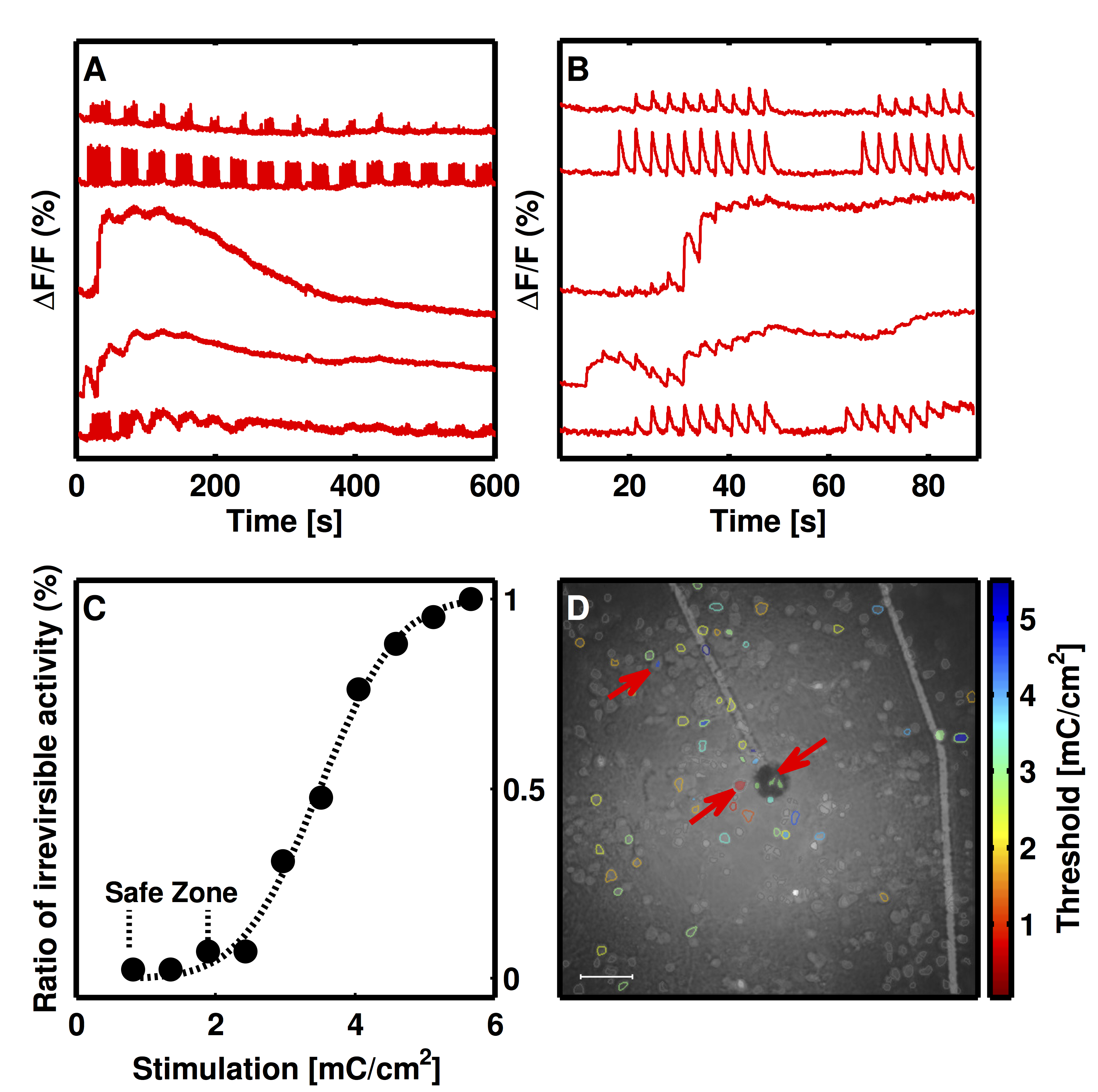

Supplement: S2 Fig — Safety mapping of electrical activation. A. Neuronal Ca2+ traces. Top two cells show stable activation, bottom three cells exhibit non-reversible activity. B. larger scale of same traces as in A. C, Ratio of cells exhibiting irreversible activity as a function of stimulation amplitude, indicating safe zone. D, Color coded activation map indicating irreversible activity threshold (bottom three cells in A, B marked by arrows). (TIF) [file pcbi.1003964.s002.tif]

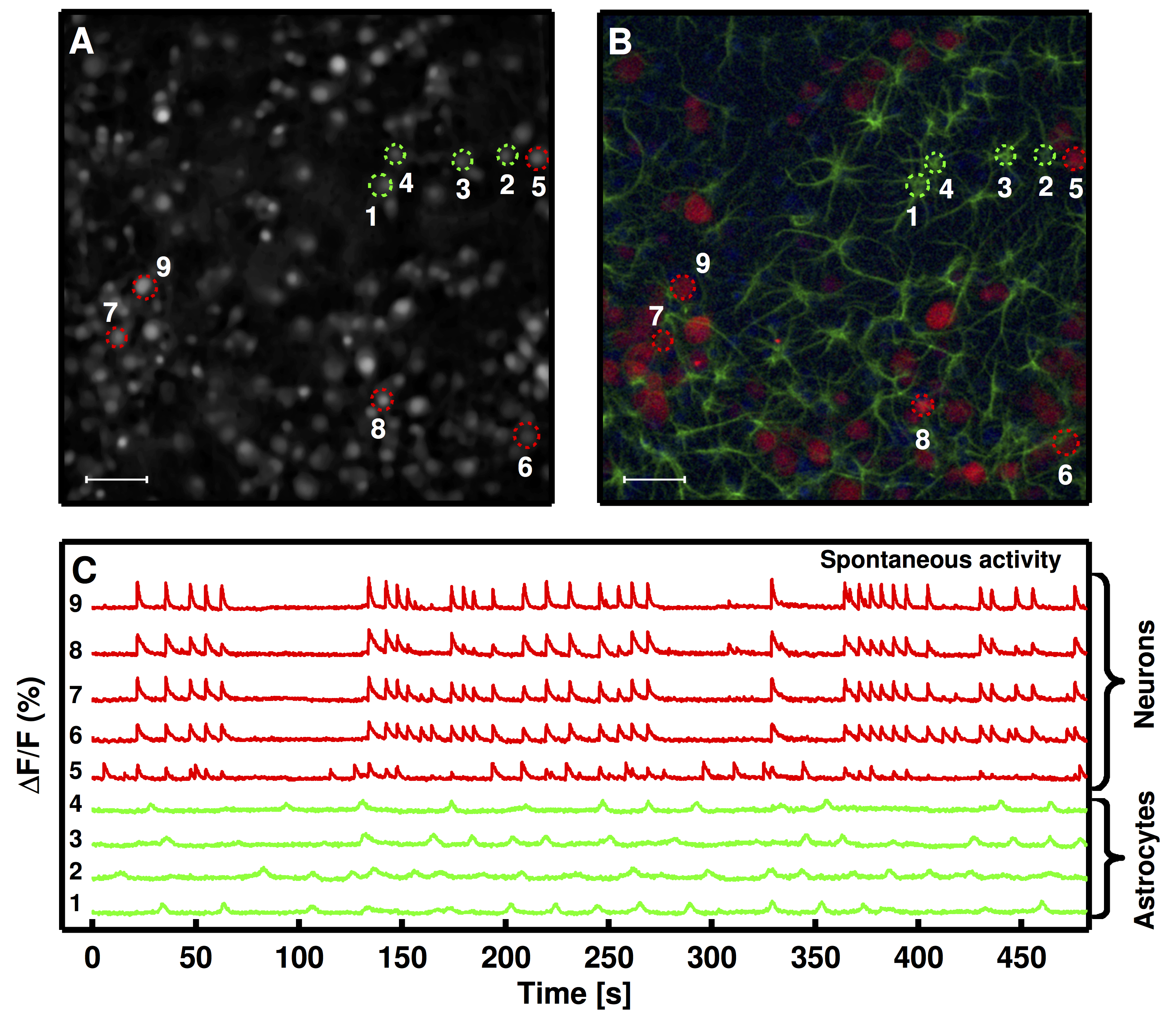

Supplement: S3 Fig — Immunostaining to distinguish neurons from astrocytes. A, Image of recorded culture with marked cells. B, Combined pseudo-color immunostaining image of same field of view and marked cells as shown in A. Red - neuronal marker NeuN. Green – astrocytic marker GFAP. Blue – nuclei visualization agent DAPI. C, Spontaneous Ca2+ traces of same neurons and astrocytes (in red and green respectively) as marked in A, B. Scale bars are 75 µm. Culture was 14 DIV. (TIF) [file pcbi.1003964.s003.tif]

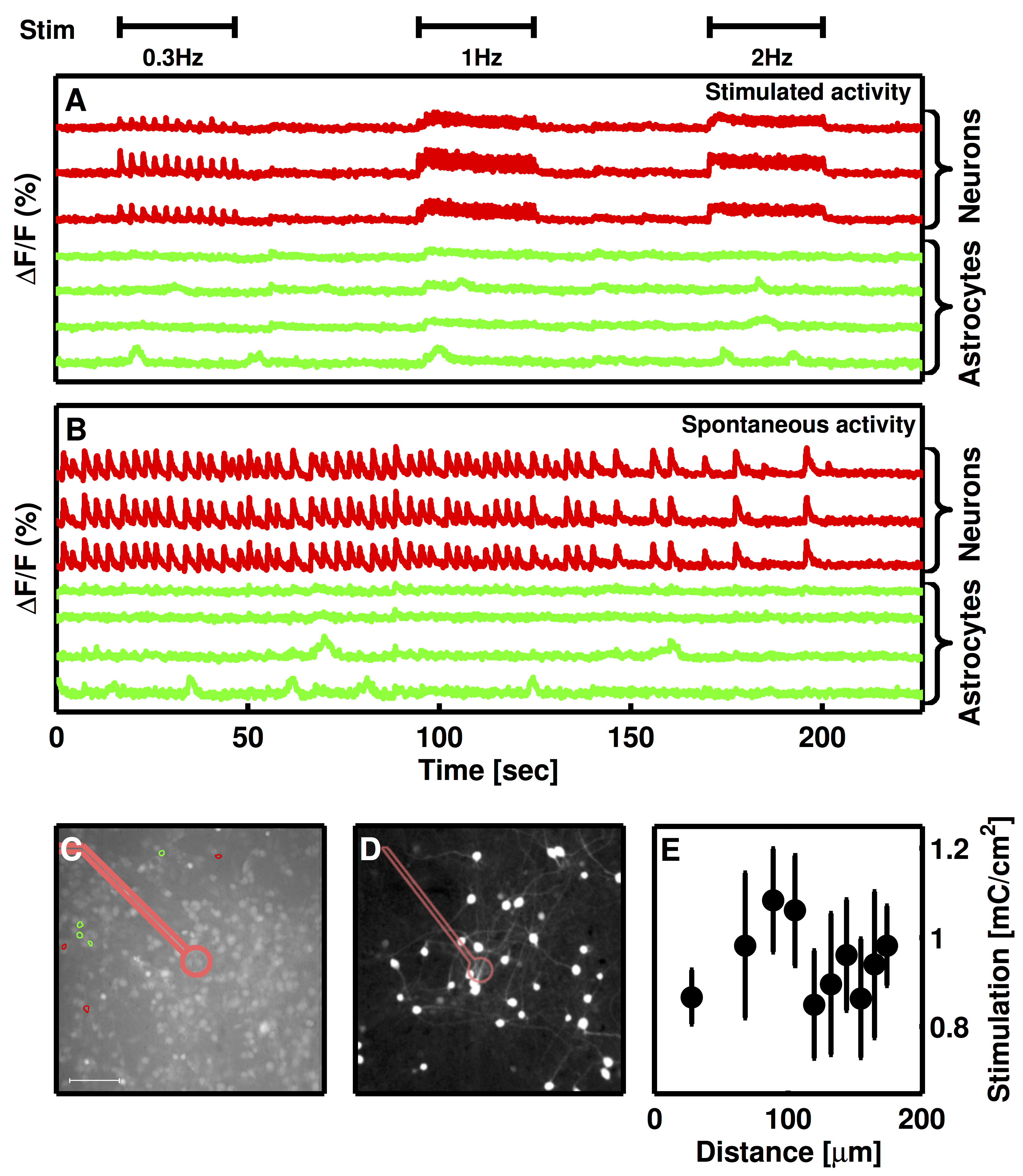

Supplement: S4 Fig — Parameters of neuronal activation. A. Ca2+ traces of typical stimulated activity. Neurons (in red) respond to electrical stimulation, and astrocytes (in green) exhibit some [Ca2+]i elevation. B. Spontaneous activity traces of same cells as in A. C, Image of recorded culture with specified cells from A, B. Electrode is marked in red, and scale bar indicates 75 µm. Culture was 17 DIV. D, Distribution of the neurites of activated cells may be delineated by stimulation triggered averaging. Density of activated neurites is highest in the vicinity of the electrode. E, Stimulation threshold as a function of distance from electrode, indicating no clear correlation between distance from electrode and stimulation threshold (standard errors indicated). (TIF) [file pcbi.1003964.s004.tif]

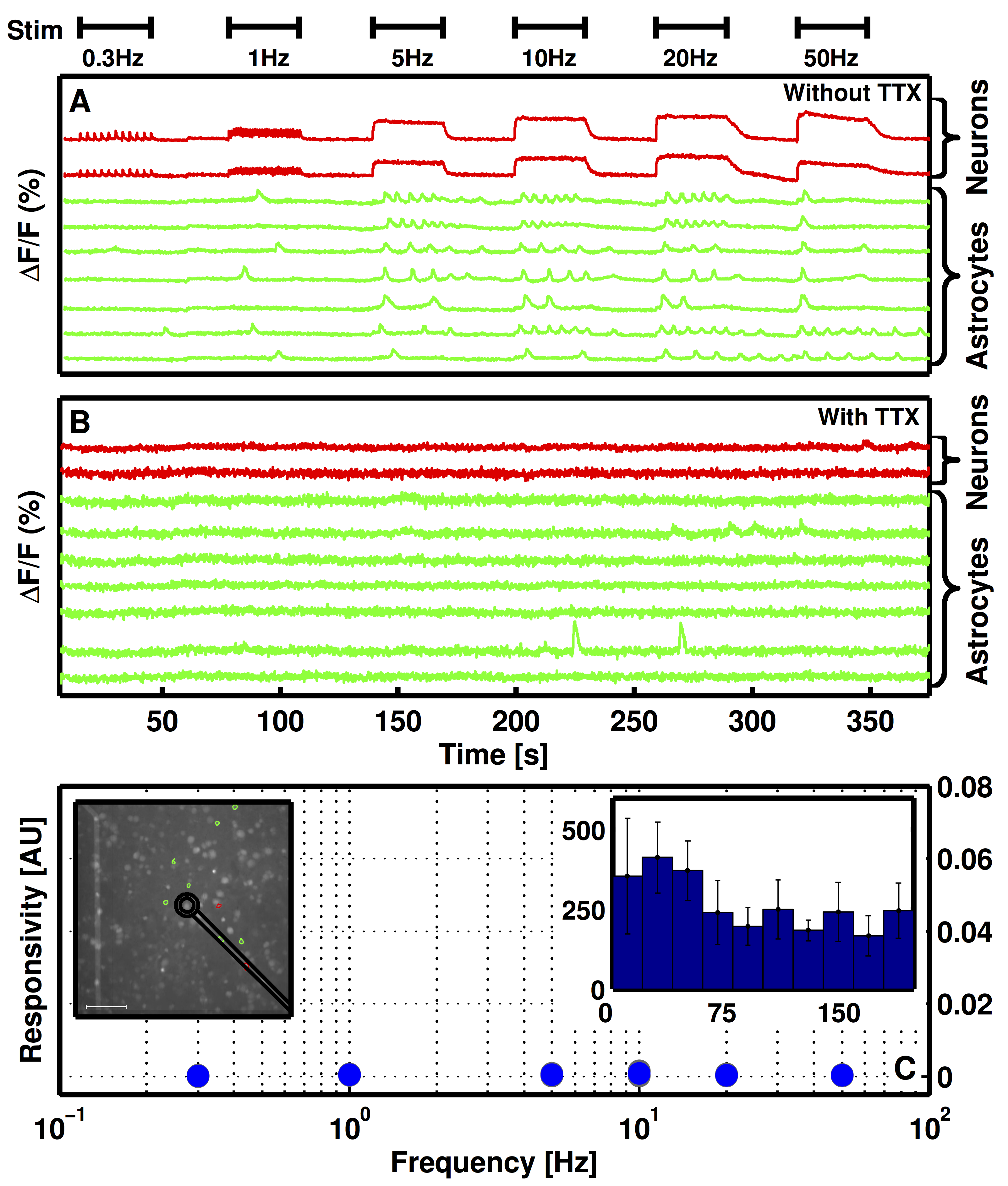

Supplement: S5 Fig — Astrocytic response is not a direct effect of electrical stimulation. A, Traces of two selected neurons (in red), showing stimulated activity according to protocol, and seven selected astrocytes (in green) in presence of neuronal AMPAR and NMDAR/kainite antagonists. B, Traces of same cells and stimulation protocol as in A, showing no neuronal and astrocytic [Ca2+]i elevations in the presence of neuronal AMPAR and NMDAR/kainite antagonists, and TTX. C, Astrocytic responsivity as a function of stimulation frequency in presence of TTX show no astrocytic frequency dependence (N = 20). Left inset shows image of recorded culture with specified cells from A, B. Electrode is marked in black, and scale bar indicates 75 µm. Right inset is a histogram of responsive astrocytes per area as a function of radius from electrode in µm (N = 277). (TIF) [file pcbi.1003964.s005.tif]

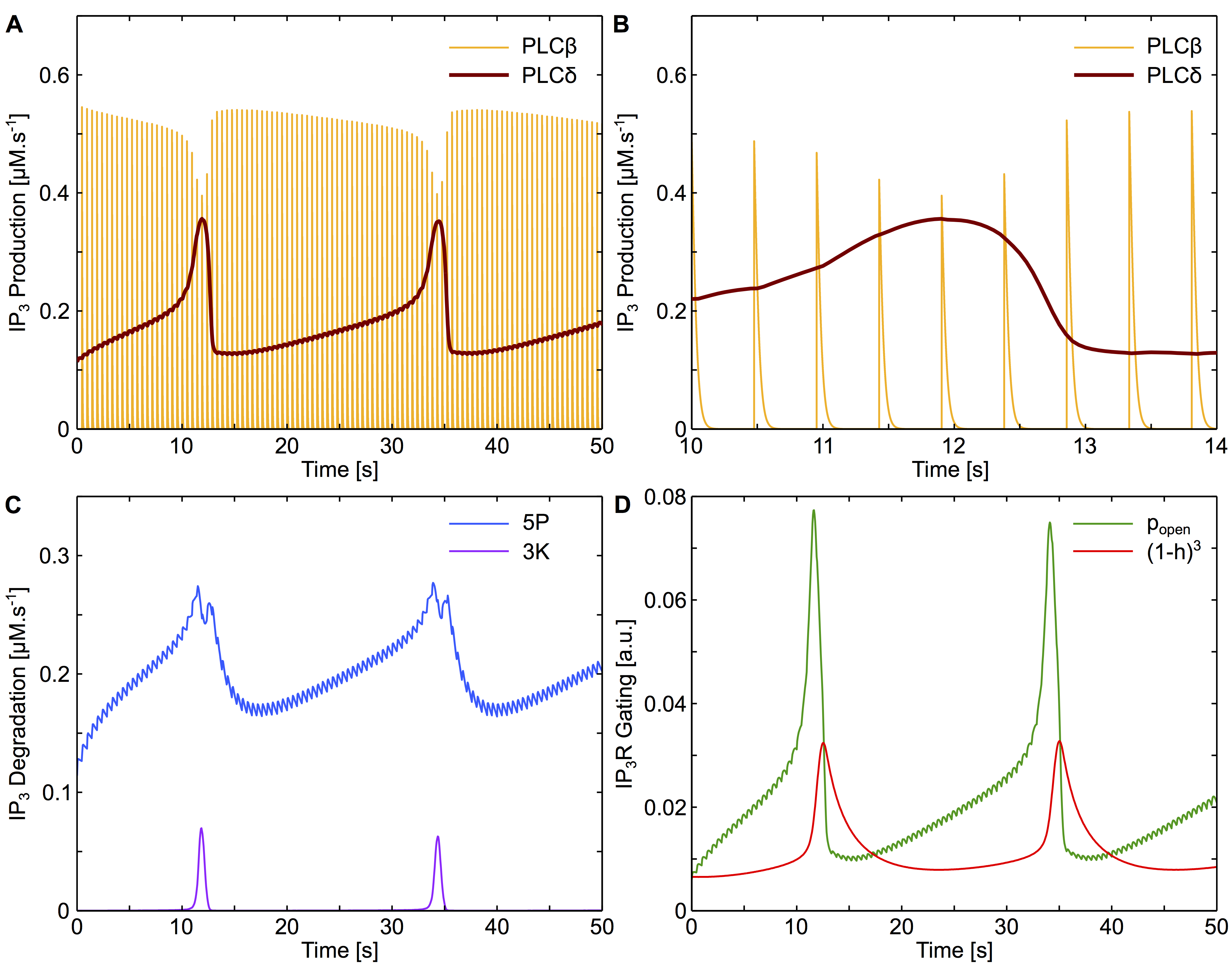

Supplement: S6 Fig — Detailed astrocytic response to a 2.1 Hz neuronal stimulation. The spiking behavior displayed by isolated astrocytes for stimulation frequency above 2 Hz (see Fig. 8) can be understood by examining the dynamics of the underlying signaling pathway. A, Above 2 Hz, the IP3 produced by PLC-β (in orange) leads to small opening of IP3R channels, increasing the Ca2+ concentration in the cytosol. This increased Ca2+ level activates PLC-δ IP3 production (in brown). B, Detailed view of PLC-β and PLC-δ IP3 production during a Ca2+ rise. C, This positive feedback loop triggers the CICR by increasing the opening probability of IP3R channels (in green). Further increases in Ca2+ inactivates IP3R channels (in red) thus ending the Ca2+ rise as it gets reintegrated into the ER. D, During this process, IP3 is degraded by Ca2+-dependent IP3-3K enzymes during the Ca2+ rise (in purple), and by Ca2+-independent IP-5P enzymes (in blue). (TIF) [file pcbi.1003964.s006.tif]
