# Supplementary material for: Glutamate Mediated Astrocytic Filtering of Neuronal Activity
Source: PLoS Comput Biol. 2014 Dec 18;10(12):e1003964. doi: 10.1371/journal.pcbi.1003964 (PMC4270452; doi:10.1371/journal.pcbi.1003964)
Supplement: S1 Table — Model parameters. (PDF) [file pcbi.1003964.s007.pdf]

Table S1. Biochemical parameters of the GChI model.

Symbol	Description	Value	Units
	IP_3R kinetics		
d_1	IP_3 binding affinity	0.13	μM
O_2	Inactivating Ca^{2+} binding rate	0.62	$\mu M^{-1}s^{-1}$
d_2	Inactivating Ca^{2+} binding affinity	1.049	μM
d_3	IP_3 binding affinity (with Ca^{2+} inactivation)	0.9434	μM
d_5	Activating Ca^{2+} binding affinity	0.08234	μM
	Calcium fluxes		
C_T	Total ER Ca^{2+} content	2	μM
ρ_A	ER-to-cytoplasm volume ratio	0.185	–
Ω_C	Maximal Ca^{2+} release rate by IP_3Rs	18.56	s^{-1}
Ω_L	Maximal Ca^{2+} leak rate	0.3416	s^{-1}
O_P	Maximal Ca^{2+} uptake rate	2.7846	$\mu M\, s^{-1}$
K_P	Ca^{2+} affinity of SERCA pumps	0.05	μM
	IP_3 production		
O_δ	Maximal rate of IP_3 production by PLCδ	0.4641	$\mu M\, s^{-1}$
K_δ	Ca^{2+} affinity of PLCδ	0.1	μM
κ_δ	Inhibiting IP_3 affinity of PLCδ	1.5	μM
O_β	Maximal rate of IP_3 production by PLCβ	1.105	$\mu M\, s^{-1}$
K_G	Glutamate affinity of the receptor	1.3	μM
K_L	Ca^{2+}/PKC-dependent inhibition factor	10	μM
K_{KC}	Ca^{2+} affinity of PKC	0.6	μM
	IP_3 degradation		
Ω_{5P}	Maximal rate of IP_3 degradation by IP-5P	0.793	s^{-1}
O_{3K}	Maximal rate of IP_3 degradation by IP_3-3K	13.923	$\mu M\, s^{-1}$
K_D	Ca^{2+} affinity of IP_3-3K	1	μM
K_{3K}	IP_3 affinity of IP_3-3K	0.7	μM
	IP_3 diffusion		
F	GJC IP_3 permeability	3.64	s^{-1}
I_θ	Threshold IP_3 gradient for diffusion	0.15	μM
ω_I	Scaling factor of diffusion	0.05	μM
	Synapse dynamics		
Ω_f	Rate of synaptic facilitation	2	s^{-1}
Ω_d	Rate of recovery of released synaptic vesicles	1	s^{-1}
Ω_G	Glutamate clearance rate	60	$\mu M\, s^{-1}$
U_0	Basal probability of synaptic glutamate release	0.25	–
ρ_C	Volume ratio of synaptic vesicles and mixing volume	6.5×10^{-4}	–
G_T	Total glutamate content of readily releasable vesicles	200	mM
ρ_{so}	Fraction of glutamate that spills over to the astrocyte	0.075	–
