# Supplementary material for: Glutamate Mediated Astrocytic Filtering of Neuronal Activity
Source: PLoS Comput Biol. 2014 Dec 18;10(12):e1003964. doi: 10.1371/journal.pcbi.1003964 (PMC4270452; doi:10.1371/journal.pcbi.1003964)
Supplement: S1 Text — Full model description (Astrocytes dynamics; Synaptic dynamics); Supporting References. (PDF) [file pcbi.1003964.s008.pdf]

SUPPLEMENTARY MATERIAL

Glutamate Mediated Astrocytic Filtering of Neuronal Activity

Gillad Wallach[1,*], Jules Lallouette[2,3,*], Nitzan Herzog[1,4], Maurizio De Pittà[1,2,3,5], Eshel Ben-Jacob[5,6], Hugues Berry[2,3] Yael Hanein[1]
1 School of Electrical Engineering, Tel Aviv University, Tel Aviv, Israel
2 EPI Beagle, INRIA Rhône-Alpes, F-69603, Villeurbanne, France
3 LIRIS, Université de Lyon, UMR 5205 CNRS-INSA, F-69621, Villeurbanne, France
4 Department of Electrical and Electronic Engineering, University of Nottingham, Nottingham, UK
5 School of Physics and Astronomy, Tel Aviv University, Ramat Aviv, Israel
6 Center for Theoretical Biological Physics, Rice University, Houston, TX, USA
***: These authors contributed equally to this work**

S1 Astrocyte Ca^{2+} signalling with the G-ChI model

S1.1 Astrocyte dynamics

The G-ChI model that we use in this study has already been published and studied in details in [1, 2]. Here we reproduce the equations without going into too much detail. As presented in the methods section of the main text, the main equations describing each astrocyte i are:

$$\frac{\mathrm{d}}{\mathrm{d}t}C_i = J_C(C_i, h_i, I_i) + J_L(C_i) - J_P(C_i) \tag{S1}$$

$$\frac{\mathrm{d}}{\mathrm{d}t}h_i = \Omega_h(C_i, I_i)\cdot(h_\infty(C_i, I_i) - h_i) \tag{S2}$$

$$\frac{\mathrm{d}}{\mathrm{d}t}I_i = J_\delta(C_i, I_i) + aJ_\beta(C_i, G_i) - J_{3K}(C_i, I_i) - J_{5P}(I_i) \tag{S3}$$

with

$$J_C(C_i, h_i, I_i) = \Omega_C \cdot m_\infty^3 h_i^3 \cdot (C_T - (1+\rho_A)C_i) \qquad m_\infty(C_i, I_i) = \mathcal{H}(C_i, d_5)\,\mathcal{H}(I_i, d_1)$$

$$J_L(C_i) = \Omega_L \cdot (C_T - (1+\rho_A)C_i) \qquad J_P(C_i) = O_P\mathcal{H}\left(C_i^2, K_P\right)$$

$$h_\infty(C_i, I_i) = d_2\frac{I_i + d_1}{d_2(I_i+d_1) + (I_i+d_3)C_i} \qquad \Omega_h(C_i, I_i) = \frac{O_2 d_2(I_i+d_1) + O_2(I_i+d_3)C_i}{I_i+d_3}$$

$$J_\delta(C_i, I_i) = O_\delta \cdot \frac{\kappa_\delta}{\kappa_\delta + I_i}\mathcal{H}\left(C_i^2, K_\delta\right) \qquad J_\beta(C_i, I_i) = O_\beta \cdot \mathcal{H}\left((\rho_{so}G)^{0.7}, K_G + K_L \cdot \mathcal{H}(C_i, K_{KC})\right)$$

$$J_{3K}(C_i, I_i) = O_{3K}\cdot\mathcal{H}\left(C_i^4, K_D\right)\mathcal{H}(I_i, K_3) \qquad J_{5P}(I_i) = \Omega_{5P}\cdot I_i$$

where the function $\mathcal{H}(x^n, K)$ denotes the sigmoid (Hill) function $\frac{x^n}{x^n+K^n}$. The values of the parameters in the above equations are given in Table **??**. In equation (S3), $a = 1$ if the astrocyte is stimulated and 0 otherwise.

S1.2 Synaptic dynamics

Astrocytes are stimulated by an equivalent synapse which represents all active synapses enwrapped by one astrocyte. This equivalent synapse is described with the Tsodyks-Markram model of dynamical synapse [3]. Glutamate concentration G in the synaptic cleft was modeled following [2]. The corresponding

equations read:

$$\frac{\mathrm{d}}{\mathrm{d}t}u = -\Omega_f u + U_0 \sum_j (1-u)\delta(t-t_j) \tag{S4}$$

$$\frac{\mathrm{d}}{\mathrm{d}t}x = -\Omega_d(1-x) - \sum_j ux\delta(t-t_j) \tag{S5}$$

$$\frac{\mathrm{d}}{\mathrm{d}t}G = -\Omega_G G + \rho_C G_T \sum_j u(t_j^+)x(t_j^-)\delta(t-t_j) \tag{S6}$$

where t_j^+ and t_j^- respectively denote the time just after and just before the presynaptic spike occuring at time t_j.

References

1. Pittà MD, Goldberg M, Volman V, Berry H, Ben-Jacob E (2009) Glutamate regulation of calcium and IP3 oscillating and pulsating dynamics in astrocytes. J Biol Phys 35: 383–411.

2. De Pittà M, Volman V, Berry H, Ben-Jacob E (2011) A tale of two stories: Astrocyte regulation of synaptic depression and facilitation. PLoS Comput Biol 7: e1002293.

3. Tsodyks MV, Markram H (1997) The neural code between neocortical pyramidal neurons depends on neurotransmitter release probability. Proc Natl Acad Sci U S A 94: 719–723.
